# Multiple interactions recruit BLTP2 to ER-PM contacts to control plasma membrane dynamics

**DOI:** 10.1083/jcb.202504027

**Published:** 2025-09-03

**Authors:** Anbang Dai, Peng Xu, Chase Amos, Kenshiro Fujise, Yumei Wu, Han Yang, Julia N. Eisen, Andrés Guillén-Samander, Pietro De Camilli

**Affiliations:** 1 Department of Neuroscience, Yale University School of Medicine, New Haven, CT, USA; 2 Department of Cell Biology, Yale University School of Medicine, New Haven, CT, USA; 3 Program in Cellular Neuroscience, Neurodegeneration and Repair, Yale University School of Medicine, New Haven, CT, USA; 4 Howard Hughes Medical Institute, Chevy Chase, MD, USA

## Abstract

BLTP2/KIAA0100, a bridge-like lipid transfer protein, was reported to localize at contacts of the ER with either the plasma membrane (PM) or recycling tubular endosomes depending on the cell type. Our findings suggest that mediating bulk lipid transport between the ER and the PM is a key function of this protein, as BLTP2 tethers the ER to tubular endosomes only after they become continuous with the PM and that it also tethers the ER to macropinosomes in the process of fusing with the PM. We further identify interactions underlying binding of BLTP2 to the PM, including phosphoinositides, the adaptor proteins FAM102A/FAM102B, and N-BAR domain proteins at membrane-connected tubules. The absence of BLTP2 results in the accumulation of intracellular vacuoles, many of which are connected to the PM, pointing to a role of the lipid transport function of BLTP2 in the control of PM dynamics.

## Teaser

Dai et al. report the localization of BLTP2 in different human cell types and reveal binding partners that mediate its tethering to the PM and to PM-connected structures, including tubular recycling endosomes and recycling macropinosomes undergoing fusion with the PM.

## Introduction

Cellular life implies continuous intracellular fluxes of lipids. This is achieved both by vesicular transport and by protein-mediated lipid transport. Until a few years ago, most lipid transport proteins were thought to function via a shuttle mechanism in which lipid-binding proteins extract lipids from a membrane and transport them piecemeal to another membrane, often in a counter-transport reaction whereby two different lipids are exchanged between the two membranes (Wong et al., 2019; Prinz et al., 2020; Reinisch et al., 2025). Recently, evidence for another mechanism of lipid transport between membranes has been reported, mediated by proteins that directly bridge two different membranes and contain a hydrophobic groove spanning their entire length along which lipids can slide from one membrane to another (Leonzino et al., 2021; Levine, 2022; Neuman et al., 2022b; Toulmay et al., 2022). These proteins, collectively referred to as bridge-like lipid transfer proteins (BLTPs), are optimally suited for fast, high-capacity lipid transport. Functions assigned to these proteins include growth of new membranes, expansion of organelles not connected to other membranes by vesicular transport, membrane repair, and roles in the rapid remodeling of the lipid composition of membranes (Hanna et al., 2023).

BLTP2/KIAA0100 is one such protein with orthologs in all eukaryotic species (Levine, 2022; Neuman et al., 2022b; Toulmay et al., 2022). It is a large protein predicted to have a rod-like structure with an N-terminal transmembrane helix that anchors it in the ER. In mammals, its absence results in embryonic lethality; in flies, where it was formerly referred to as “hobbit,” it is essential for development and synaptogenesis (Neuman and Bashirullah, 2018; Neuman et al., 2024, *Preprint*), and in plants, for root growth and cytokinesis (Cheng and Bezanilla, 2021). Genetic studies of human BLTP2 and of its orthologs in yeast (Fmp27 and Ypr117w) and in worms (F31C3.3) have suggested its role in the adaptation of life to lower temperature (Banerjee et al., 2025; Toulmay et al., 2022), most likely via their property to rapidly modify the membrane lipidome to ensure normal membrane fluidity at reduced temperature (known as homeoviscous adaptation). A similar function has been assigned to BLTP1 in yeast (Csf1) (John Peter et al., 2022a; John Peter et al., 2022b; Toulmay et al., 2022) and worms (LPD-3) (Pandey et al., 2023; Wang et al., 2022). Concerning its site(s) of action within cells, studies in yeast, *Drosophila* cells, and a human cancer cell line reported its concentration at ER-plasma membrane (PM) contacts (Banerjee et al., 2025; Neuman et al., 2022a; Toulmay et al., 2022), while another study of mammalian cell lines reported its localization at contacts between the ER and tubular recycling endosomes (Parolek and Burd, 2024), rather than at ER-PM contacts. A localization of yeast Fmp27 and Ypr117w at some ER-mitochondria contacts was also reported (Toulmay et al., 2022).

The goal of this study was to advance our understanding of the localization and function of BLTP2. We consistently found BLTP2 at contacts between the ER and the PM, although in different patterns depending on the cell line. In cells where BLTP2 was localized at contacts of the ER with apparently internal membranes, such as tubular endosomes or macropinosomes, we found that these membranes were continuous with, or in the process of becoming continuous with, the PM. We also identified interactions responsible for these localizations, including the binding of BLTP2 to two PM adaptors, FAM102A and FAM102B, whose yeast ortholog (Hoi1/Ybl086c) was independently identified as a binding partner of the yeast BLTP2 orthologs (Fmp27 and Ypr117w) by Elizabeth Conibear and co-workers (Dziurdzik et al., 2025). Finally, we found that cells lacking BLTP2 have striking alterations of their internal structure with the abundant presence of intracellular vacuoles positive for PM markers and open to the cell surface. These alterations reveal a function of BLTP2 in controlling the dynamics of the PM, which may result from a defect in BLTP2-dependent lipid transport to this membrane.

## Results

### BLTP2 localizes at contact of the ER with the PM, including deep PM invaginations continuous with tubular recycling endosomes

**Figure 1.**
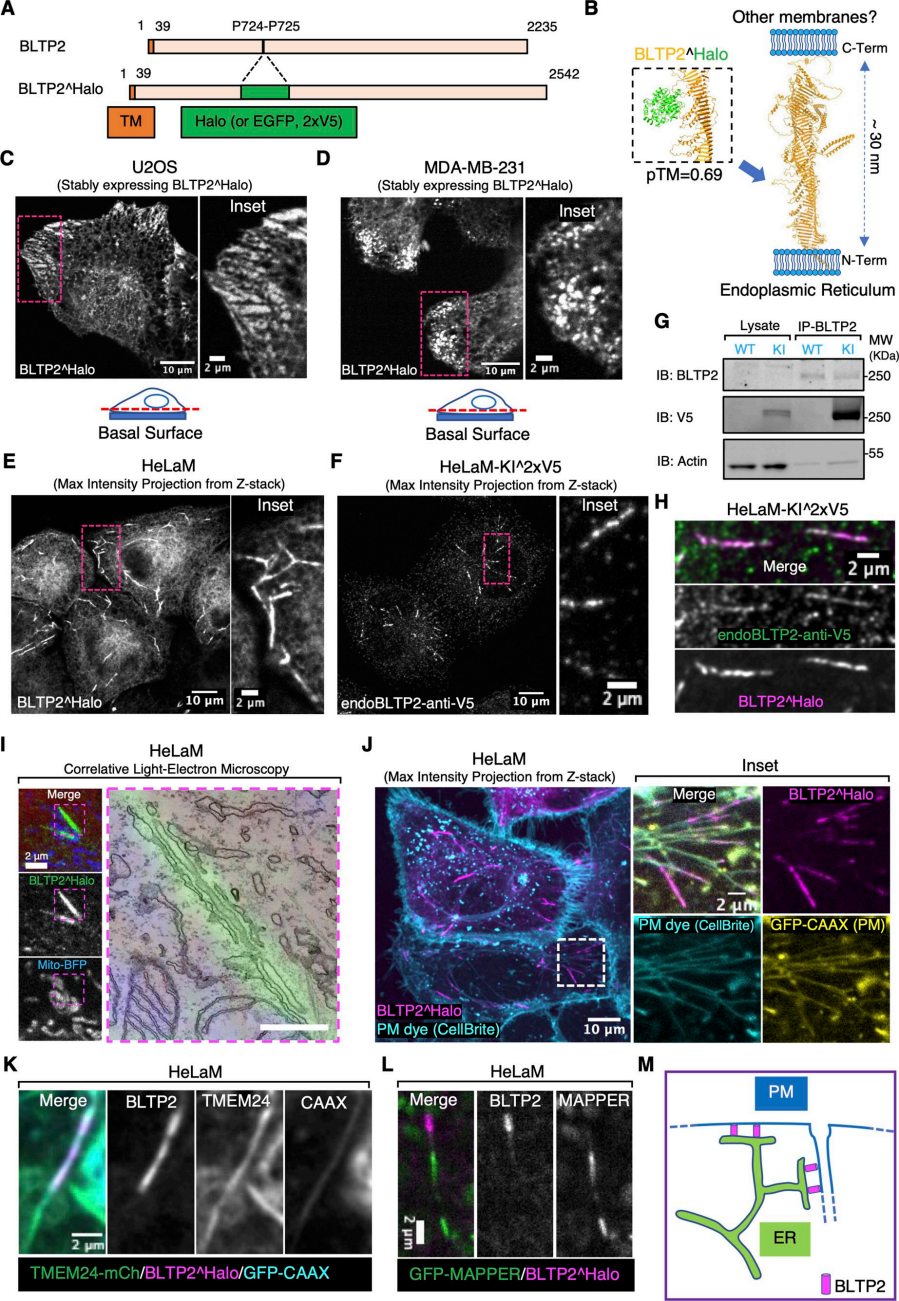
**BLTP2 is enriched at ER contacts with the PM and PM-connected tubular internal membranes. (A)** Domain diagram of human BLTP2 and of the internally tagged (Halo, EGFP, or 2xV5 epitopes) BLTP2. TM indicates the transmembrane region of BLTP2. **(B)** Schematic model of the arrangement of BLTP2 at contacts of the ER with other membranes. The arrow indicates the site where tags (Halo, EGFP, or 2xV5 epitopes) were inserted. Structures are predicted using AlphaFold3. **(C and D)** U2OS cells (C) and MDA-MB-231 cells (D) stably expressing BLTP2^Halo show enrichment of this protein at ER-PM contacts near the edge of the cell. An optical section close to the basal surface (see dashed red line in the cartoon) is shown. **(E)** HeLaM cells stably expressing BLTP2^Halo show enrichment of the protein at tubular structures. **(F)** Localization of endogenous BLTP2 in gene-edited HeLaM cells where the 2xV5 epitope was inserted in the coding sequence of BLTP2. Anti-V5 immunofluorescence reveals enrichment of BLTP2 on tubular structures. **(G)** Validation of the endogenous tagging of BLTP2 by western blotting. Anti-BLTP2 immunoprecipitation co-enriched a V5 immunoreactive band. **(H)** Endogenous BLTP2 (V5 immunoreactivity) co-localizes with exogenous BLTP2^Halo on the same tubular structures. **(I)** CLEM revealed that a BLTP2-positive tubular structure represents a tubular membrane surrounded by ER. Scale bar, 500 nm. **(J)** BLTP2^Halo localizes at the distal portion of tubular structures that are positive for the PM marker GFP-CAAX and are labeled by the membrane-impermeant PM dye CellBrite. **(K)** TMEM24-mCherry, another ER-PM contact protein, is also present on BLTP2-positive tubular structures but only partially co-localizes with BLTP2^Halo. **(L)** GFP-MAPPER, an artificial ER-PM tethering protein, is also present on BLTP2-positive tubular structures but does not overlap with BLTP2^Halo. **(M)** Schematic drawing of BLTP2 localization at contacts of the ER with both the outer PM and the distal portions of PM-connected tubular structures. Source data are available for this figure: SourceData F1.

To investigate the cellular localization of BLTP2 in human cells, we first expressed, transiently or constitutively through a lentivirus, BLTP2 constructs with an internal fluorescent tag (BLTP2^Halo or BLTP2^EGFP) (Fig. 1 A) in different cell types (U2OS cells, MDA-MB-231 cells, HeLaM cells, and COS-7 cells). The tag was inserted in a loop emerging from its predicted rod-like core at a site not predicted to affect BLTP2 folding based on AlphaFold3 (Abramson et al., 2024) (Fig. 1 B), as the C-terminal region of BLTP2 had been implicated in tethering ER-anchored BLTP2 to other membranes in *Drosophila* cells (Neuman et al., 2022a). Thus, we wished to avoid perturbation of this interaction, which appears to be critical for BLTP2 function.

**Figure S1.**
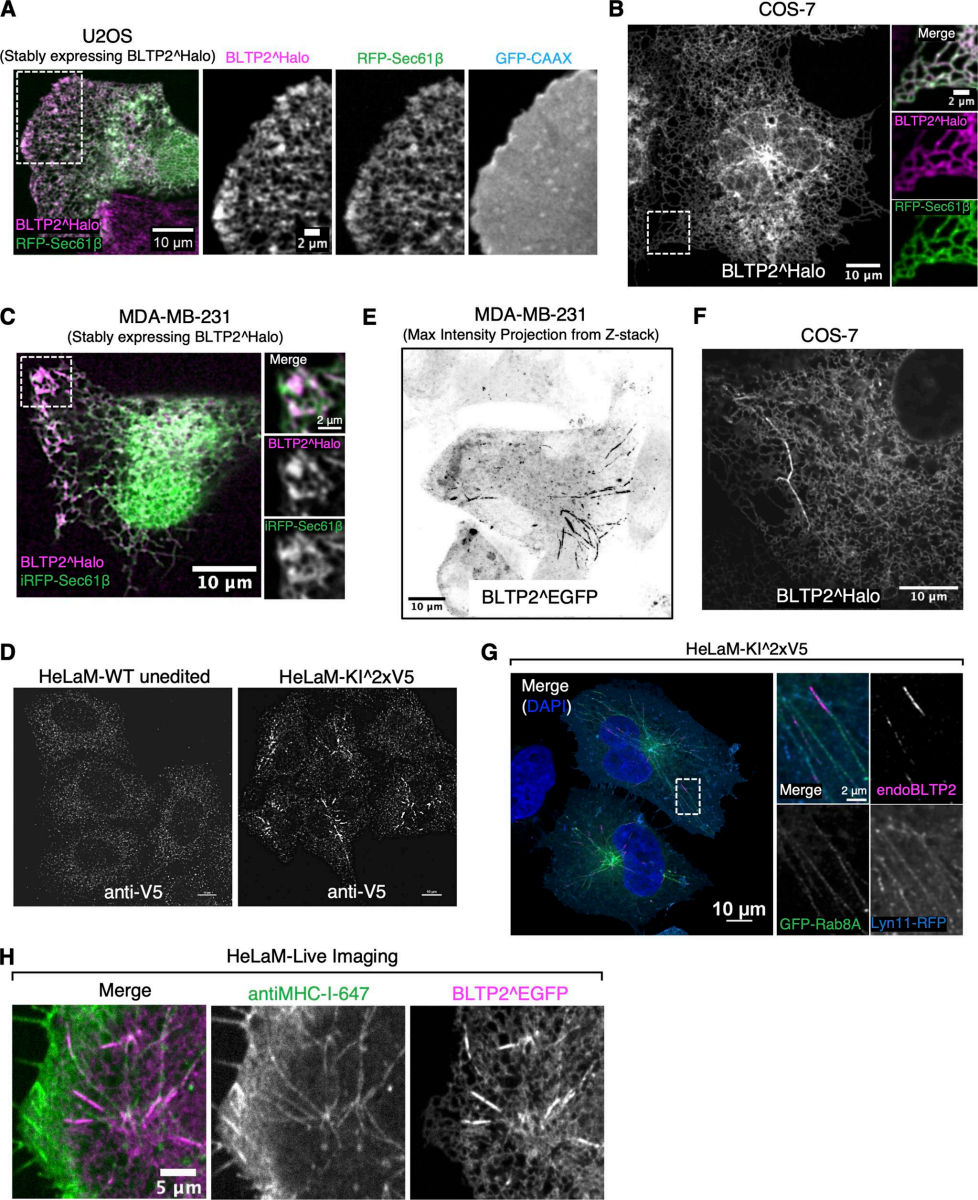
**BLTP2^Halo shows heterogeneous localization in different cell types. (A–C)** Localization of BLTP2^Halo in U2OS cells (A), COS-7 cells (B), and MDA-MB-231 cells (C). BLTP2^Halo co-localizes throughout the ER with ER marker Sec61β in all these cells. In U2OS and MDA-MB-231 cells, it shows an additional enrichment at ER-PM contacts. **(D)** Small puncta of anti-V5 immunoreactivity are present both in unedited HeLaM cells and in HeLaM-KI^2XV5, showing that such staining is nonspecific. **(E)** In some MDA-MB-231 cells, BLTP2^Halo shows an enrichment on tubular structures similar to those observed in HeLaM cells. **(F)** BLTP2^Halo mainly localizes throughout the ER in COS-7, and only occasionally it is enriched at some tubular structures. **(G)** BLTP2-positive tubules in HeLaM cells are positive for Rab8A. **(H)** A fluorescently labeled MHC-I antibody added to HeLaM cells expressing BLTP^EGFP labels the tubules.

Consistent with BLTP2 being a resident integral membrane protein of the ER (Neuman and Bashirullah, 2018; Neuman et al., 2022a; Parolek and Burd, 2024; Toulmay et al., 2022), tagged BLTP2 produced a diffuse ER fluorescence, as revealed by the co-expression of the ER marker RFP (or iRFP)-Sec61β (Fig. S1, A–C). However, in addition to this diffuse ER fluorescence, focal accumulations of BLTP2^Halo were observed with cell line–specific differences. In U2OS and MDA-MB-231 cells, BLTP2-positive patches with the typical appearance of ER-PM contacts were visible, primarily at the basal surface of these cells and concentrated at the leading edge in migrating cells (Fig. 1, C and D; and Fig. S1, A and C). In HeLaM cells such patches were not visible, and BLTP2^Halo was instead concentrated at the distal portion of tubular internal structures directed toward the cell periphery (Fig. 1 E). This localization reflected the native localization of BLTP2 in these cells, as it overlapped with anti-V5 immunofluorescence of gene-edited HeLaM cells harboring a twin-V5 epitope tag in endogenous BLTP2 (at the same internal site used to tag with Halo exogenous BLTP2) (Fig. 1, F–H). The scattered spots of anti-V5 fluorescence not positive with BLTP2^Halo represented nonspecific staining, as they were also observed in unedited WT cells (Fig. S1 D). Similar BLTP2-positive tubules were only occasionally observed in MDA-MB-231 cells (Fig. S1 E). In COS-7 cells, BLTP2 hot spots, either in the form of tubules (as in HeLaM cells), or of small patches at the cell periphery, were only infrequently observed (Fig. S1 F).

**Figure 2.**
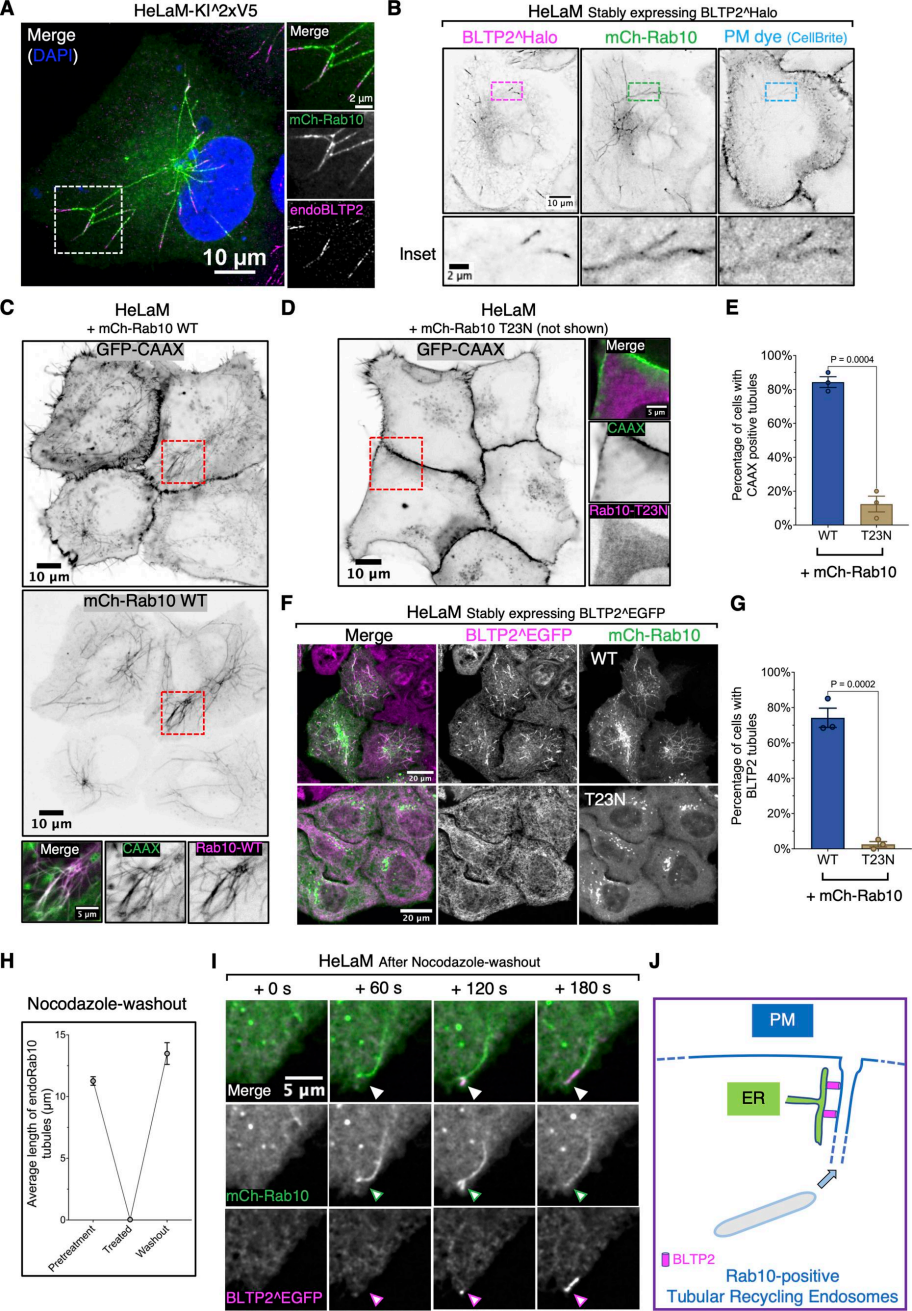
**BLTP2-positive tubular internal membranes are Rab10-dependent tubular recycling endosomes continuous with the PM. (A)** Endogenous BLTP2 localizes at the tip of mCherry-Rab10–positive tubular endosomes in HeLaM cells. **(B)** BLTP2^Halo- and mCherry-Rab10–positive tubular endosomes in HeLaM cells are connected with the PM as revealed by labeling with the membrane-impermeant PM dye CellBrite. **(C)** mCherry-Rab10–positive tubular endosomes in HeLaM cells are also labeled with the PM marker GFP-CAAX. **(D and E)** Absence of GFP-CAAX–positive tubules in HeLaM cells expressing dominant-negative Rab10 (mCh-Rab10 T23N). Fluorescence image in D and quantification in E. Two-tailed *t* test. Mean ± SEM. *n* = 3 independent experiments. 48 cells for WT and 57 cells for the T23N mutant. **(F and G)** Expression of dominant-negative Rab10 (mCh-Rab10 T23N) abolished BLTP2^EGFP-positive tubules. Fluorescence images in F and quantification in G. Two-tailed *t* test. Mean ± SEM. *n* = 3 independent experiments. 127 cells for WT Rab10 and 121 cells for the T23N mutant. **(H)** Tubular structures positive for endogenous Rab10 immunoreactivity disappear after nocodazole treatment for 2 h but are restored after washing out the drug. *n* = 3 independent experiments. Pretreatment: 269 Rab10 tubules from 28 cells; treated: zero tubules observed in the 46 cells examined; washout: 353 Rab10 tubules from 26 cells. **(I)** Live imaging of a HeLaM cells after nocodazole washout shows the recovery of a Rab10-positive tubular endosome and the recruitment of BLTP2^EGFP after the tubule reaches the PM and fuses with it. **(J)** Schematic drawing of BLTP2 recruitment and localization at a Rab10-positive tubular endosome that is continuous with the PM.

**Figure S2.**
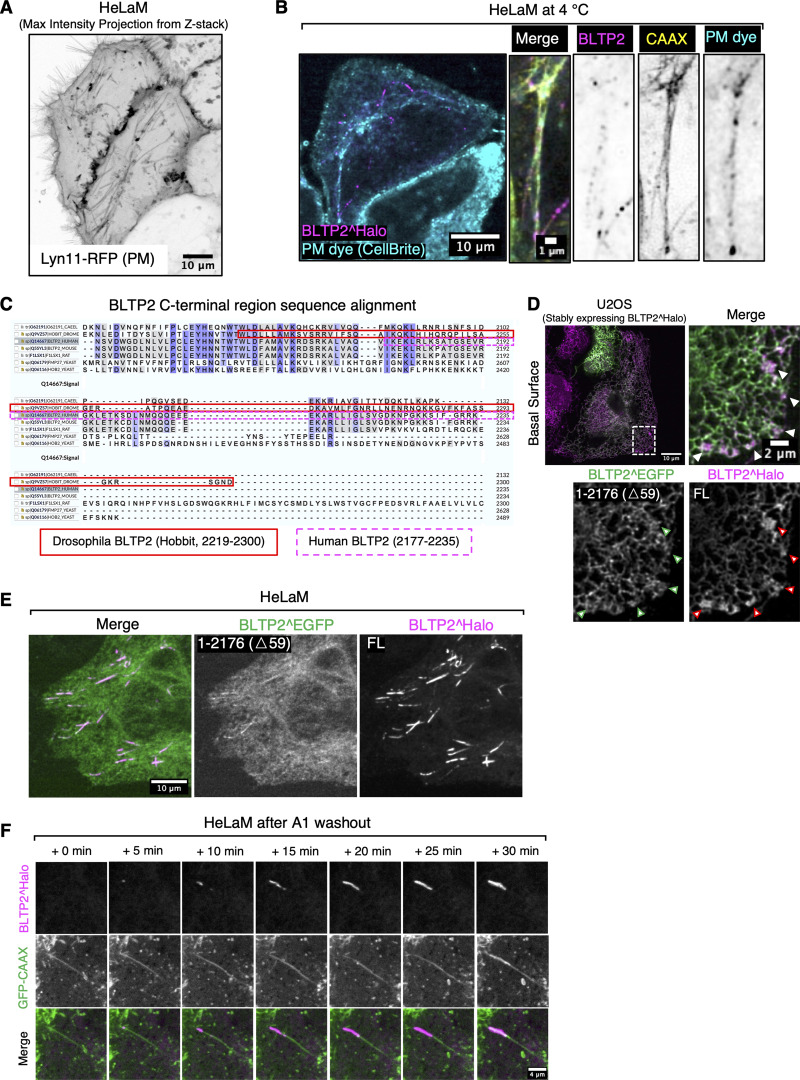
**BLTP2-positive tubular endosomes are positive for PM markers, and its C- terminal region is required for its PI4P-dependent tethering function. (A)** The tubular structures of HeLaM cells are also positive for another PM marker, Lyn11-RFP. **(B)** BLTP2-positive tubules are also labeled by CellBrite at 4°C. **(C)** Sequence alignment of the C-terminal region of human BLTP2 with the corresponding region of other species. Conserved residues are highlighted in blue, with darker color indicating higher conservation. aa 2219–2300 of *Drosophila* BLTP2 (hobbit) are framed by red solid lines, and aa 2177–2235 of human BLTP2 by magenta dashed lines. **(D and E)** U2OS cells (D) and HeLaM cells (E) co-expressing the BLTP2 C-terminal deletion mutant BLTP2^EGFP(△59) and BLTP2^Halo FL. BLTP2^EGFP(△59) shows reduced localization relative to BLTP2^Halo FL at ER-PM contact sites in U2OS cells (arrowhead) and at tubular structures in HeLaM cells. **(F)** BLTP2 re-establishes contact with tubular endosomes after A1 washout.

The selective concentration of both exogenous and endogenous BLTP2 on tubular structures positive for Rab8 and Rab10 in HeLa cells was previously reported and thought to reflect contacts between the ER and tubular recycling endosomes (Parolek and Burd, 2024). In agreement with this previous study, we detected the presence of Rab8 and Rab10 on the BLTP2-positive tubules in HeLaM cells (Fig. S1 G and Fig. 2 A). Moreover, the addition of antibodies directed against the ectodomain of the major histocompatibility complex class I (MHC-I) complex to live HeLaM cells expressing BLTP2^EGFP resulted not only in a diffuse fluorescence of the cell surface, where the bulk of MHC-I is localized, but also in the labeling of the BLTP2-positive tubules (Fig. S1 H), in agreement with the known presence of these complexes in tubular endosomes (Caplan et al., 2002; Naslavsky et al., 2004; Radhakrishna and Donaldson, 1997). We confirmed by correlative-light EM (CLEM) that BLTP2-positive linear structures corresponded to contacts between the ER and membrane tubules (Fig. 1 I). We found, however, that at least the great majority of BLTP2-positive tubular structures were positive for PM markers, such as GFP-CAAX (Hancock et al., 1991) or Lyn11-RFP (Inoue et al., 2005) (Fig. 1 J and Fig. S2 A). Accordingly, these tubular structures became labeled with the non-permeable membrane dye CellBrite steady 650 after a few minutes of incubation at 37°C (Fig. 1 J and Fig. 2 B) or after 15 min of incubation at 4°C (Fig. S2 B). Other ER-PM tethers, such as TMEM24-mCherry (Johnson et al., 2024) or GFP-MAPPER (Chang et al., 2013) (a synthetic ER-PM tether), were also found at the tubular structures (Fig. 1, K and L). However, these other proteins either only partially (TMEM24) co-localized, or did not (MAPPER) co-localize with BLTP2 on the tubules and were additionally concentrated at other ER-PM contacts, such as those on the basal surface of the cell, where BLTP2 was not observed. We conclude that while in HeLaM cells BLTP2 localizes at contacts with tubular internal membranes with the properties of tubular recycling endosomes, such membranes are continuous with the PM and thus represent extensions of the PM (Fig. 1 M). These tubules are likely the same as the recently described Rab10-positive tubules open to the PM (Zhu et al., 2024), which were referred to as PM invaginations rather than as recycling endosomes. The presence of BLTP2 only on the most distal (peripheral) portion of the tubules indicates that despite their continuity with the PM, a heterogeneity is maintained along them with a progressive transition from PM-like properties in their peripheral portions to *bona fide* endosomal properties toward the cell center.

### Tubular recycling endosomes acquire BLTP2-positive contacts as they fuse with the PM

The CAAX-positive tubules surrounded by BLTP2 in their distal portion were Rab10 dependent, as they were no longer observed in cells expressing dominant-negative Rab10 mutant (Rab10-T23N) (Fig. 2, C–G). Formation of Rab10-dependent tubules, in turn, is known to be driven by microtubules based motor proteins, KIF13A and KIF13B (Etoh and Fukuda, 2019). Accordingly, they were disrupted by depolymerization of microtubules using nocodazole as described (Etoh and Fukuda, 2019) but reformed starting from the central region of the cell after washout of the drug (Fig. 2 H). This property offered us the opportunity to monitor the appearance of the BLTP2 signal upon their regrowth. We found that BLTP2 was recruited at their tips only when they reached the cell periphery and became continuous with the cell surface (Fig. 2, I and J; and Video 1).

**Video 1.** **HeLaM cell expressing BLTP2^EGFP and mCherry-Rab10 showing the recovery of mCherry-Rab10–positive tubules after the wash out of nocodazole, and the recruitment of BLTP2^EGFP at their tips when they reach the PM.** 30 s intervals at 60 frames/s.

### Recruitment of BLTP2 to PM-connected tubular recycling endosomes requires PI4P generated by PI4KIIIα in their membrane

The results reported above indicate that a shared feature of the ER contact sites populated by BLTP2 is to be with the PM, in spite of cell-specific differences in their localization within this membrane. We next investigated the PM determinants responsible for these localizations.

**Figure 3.**
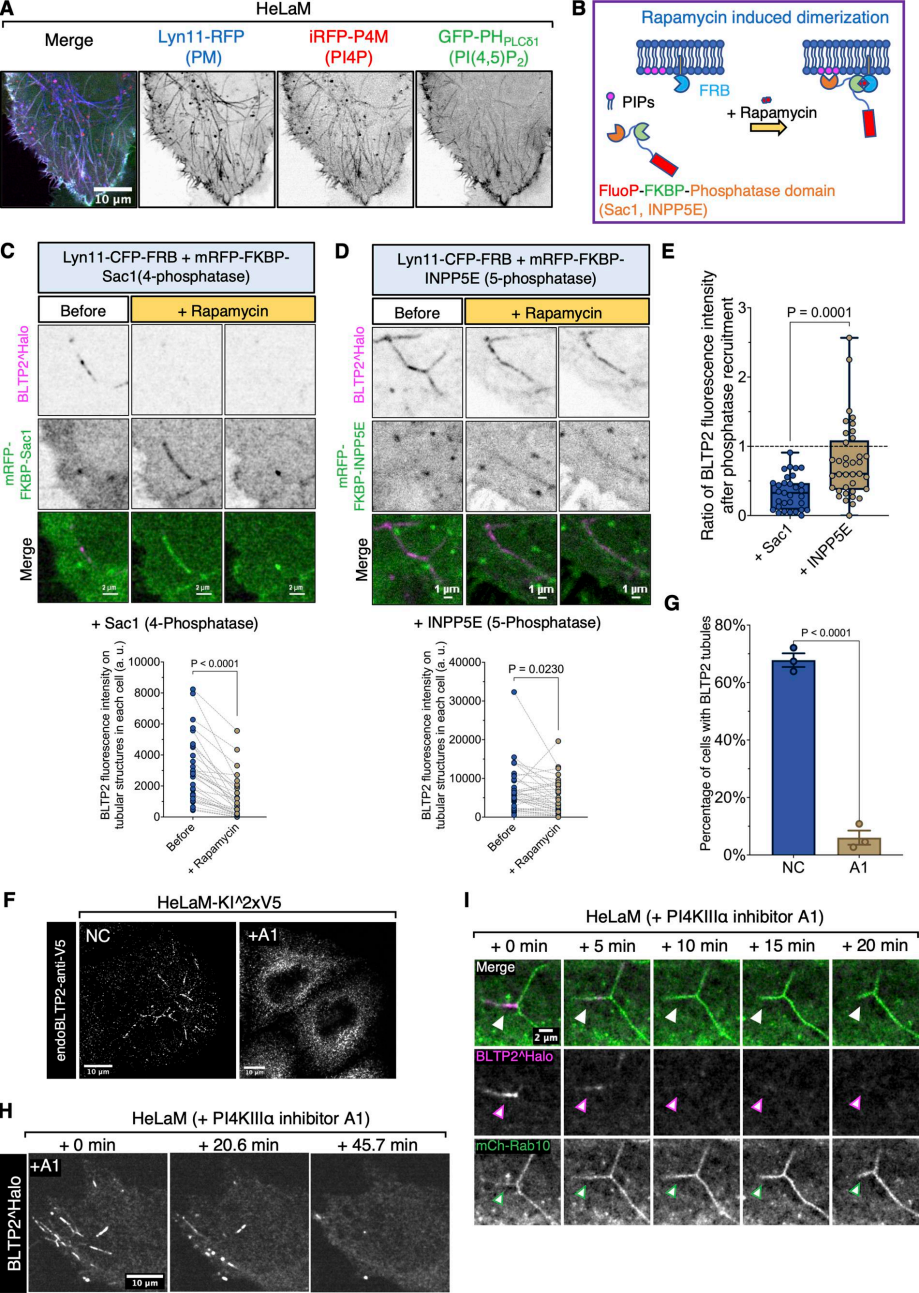
**PI4P regulates BLTP2-dependent contacts of the ER with tubular endosomes. (A)** PM-connected tubular endosomes in HeLaM cells are positive for PI4P (labeled by iRFP-P4M) and PI(4,5)P_2_ (labeled by GFP-PH_PLCδ1_) markers. **(B)** Design of the rapamycin-dependent dimerization assay to recruit the 4-phosphatase domain of Sac1 (target PI4P) or the 5-phosphatase domain of INPP5E (target PI(4,5)P_2_) to PM-connected tubular endosomes. **(C and D)** BLTP2^Halo disassociates from tubular endosomes after PI4P depletion on their membranes following the recruitment of RFP-FKBP-Sac1. In contrast, BLTP2^Halo shows no clear change after PI(4,5)P_2_ depletion following the recruitment of RFP-FKBP-INPP5E. In the quantification graphs shown at the bottom of the two panels, pairs of dots connected by a dashed line represent the total tubular BLTP2 fluorescence in each cell before and after the addition of rapamycin. Paired *t* test. Sac1: *n* = 31 cells. INPP5E: *n* = 37 cells. Data are from two independent experiments. **(E)** The loss of BLTP2 tubular fluorescence after phosphatase recruitment was quantified by dividing the total tubular fluorescence intensity in each cell after the addition of rapamycin by the value obtained before rapamycin. Points below the dashed line at y = 1 indicate a decrease of the intensity after phosphatase recruitment. Unpaired *t* test. *n* = 31 cells for Sac1 and *n* = 37 cells for INPP5E. Both are from two independent experiments. Box and whiskers indicate min to max. **(F)** Endogenous BLTP2 also undergoes disassociation from tubular endosomes in response to treatment with A1. **(G)** Cells in Fare quantified for the presence of BLTP2 tubules. Two-tailed *t* test. Mean ± SEM. *n* = 3 independent experiments. Non-treated (NC): 85 cells; A1 treated: 118 cells. **(H)** BLTP2^Halo gradually disassociates from tubular endosomes after PI4KIIIα inhibition in response to addition of the A1 compound. **(I)** mCh-Rab10 tubular endosomes persist after BLTP2^Halo disassociation from them in response to A1 treatment.

Many proteins that function as ER-PM tethers bind PI4P and PI(4,5)P_2_, two phosphoinositides concentrated in the PM (Di Paolo and De Camilli, 2006). As stated above, BLTP2 comprises a C-terminal region that contains numerous basic aa whose deletion in *Drosophila* (aa 2219–2300) abolishes its localization at ER-PM contacts and its physiological function (Neuman et al., 2022a). Accordingly, we tested the effect of the deletion of the C-terminal region in human BLTP2 and found that the removal of the last 59 aa (aa 2177–2235), which has an overall basic charge, was sufficient to reduce its concentration not only at the “outer” PM (Fig. S2, C and D), but also at the surface-connected membrane tubules (Fig. S2 E), where both PI4P and PI(4,5)P_2_ are concentrated as revealed by the expression of iRFP-P4M (Hammond et al., 2014) and GFP-PH_PLCδ1_ (Stauffer et al., 1998; Várnai and Balla, 1998), respectively (Fig. 3 A). To confirm the role of these two phosphoinositides in BLTP2 localization, we used the rapamycin-dependent FKBP–FRB heterodimerization system (Ho et al., 1996; Inoue et al., 2005) to acutely recruit phosphoinositide phosphatases to the PM to deplete them (Fig. 3 B). We co-expressed a “bait” construct comprising the PM-targeting sequence of Lyn11 followed by the FRB domain (Lyn11-CFP-FRB) and a “prey” construct comprising the FKBP domain followed either by the 4-phosphatase domain of Sac1 (to dephosphorylate PI4P to PI) (mRFP-FKBP-Sac1) (Szentpetery et al., 2010) or the 5-phosphatase domain of INPP5E (to dephosphorylate PI(4,5)P_2_ to PI4P) (mRFP-FKBP-INPP5E) (Hammond et al., 2012). Upon rapamycin addition to induce heterodimerization, robust recruitment of either prey construct from the cytosol to the tubules was observed. However, only after the recruitment of mRFP-FKBP-Sac1, a robust disassociation of BLTP2 from the tubules was observed, revealing a critical role of PI4P (Fig. 3, C–E).

PI4P localized at the PM is primarily synthesized by PI4KIIIα, the kinase encoded by the PI4KA gene (Nakatsu et al., 2012). Supporting the importance of the PM pool of PI4P in the binding of ER-anchored BLTP2 to the tubules, the addition of the A1 compound, a specific PI4KIIIα inhibitor that in the absence of triggered PI(4,5)P_2_ hydrolysis, selectively decreases PI4P but not PI(4,5)P_2_ at the PM (Bojjireddy et al., 2014; Chung et al., 2015), induced the dissociation of both endogenous BLTP2 (Fig. 3, F and G) and BLTP2^Halo (Fig. 3 H) from the tubules although the tubular network was not disrupted (Fig. 3 I). This reaction was also reversible, as washout of the drug after 1 h treatment resulted in the reformation of BLTP2-positive contacts on the tubules (Fig. S2 F and Video 2). We conclude that PI4P, but not PI(4,5)P_2_, is required for the tethering function of BLTP2 at these sites. While these results reveal the importance of PI4P in BLTP2 recruitment, PI4P is unlikely to represent the unique determinant for the concentration of BLTP2-dependent contacts at a specific PM sub-compartment. PI4P may function as an essential coreceptor. Thus, we considered the possible occurrence of protein receptors for BLTP2 in the PM.

**Video 2.** **HeLaM cell expressing BLTP2^Halo and GFP-CAAX showing recovery of BLTP2^Halo-positive contacts on a GFP-CAAX-positive tubule after the washout of the A1 compound.** The washout is carried out after A1 treatment for 1 h. 60 s intervals at 60 frames/s.

### FAM102A and FAM102B are PM adaptors for BLTP2

**Figure 4.**
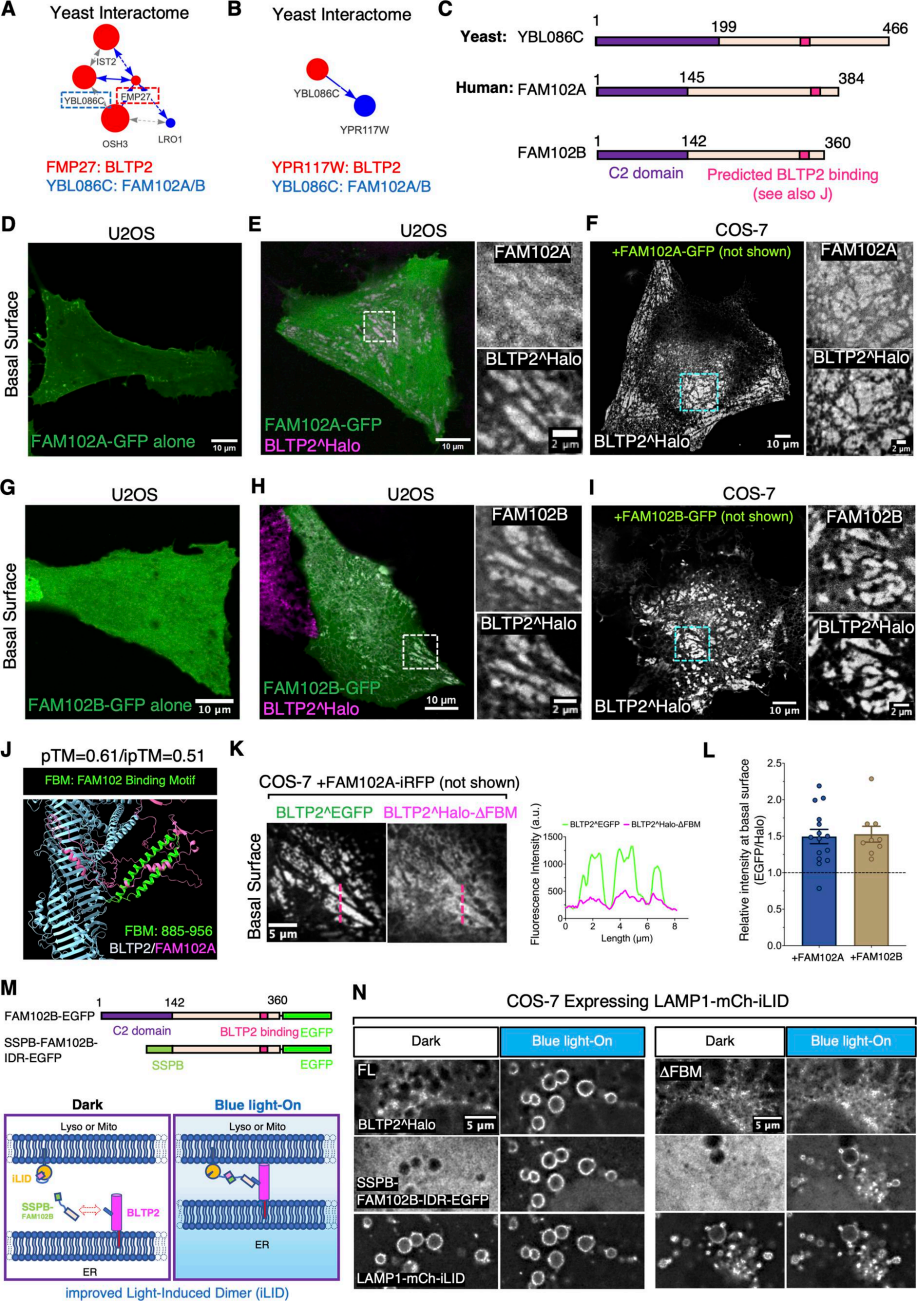
**An interaction of BLTP2 with FAM102A and FAM102B enriches BLTP2 at ER-PM contacts. (A and B)** Interaction diagram exported from the Yeast Interactome Website (https://yeast-interactome.biochem.mpg.de:3838/interactome/) revealing protein interactions of the BLTP2 yeast orthologs Fmp27 (A, score = 10) and Ypr117w (B, score = 3) with Ybl086c, the ortholog of mammalian FAM102A/B. **(C)** Domain organization of Ybl086c with FAM102A/B comprising an N-terminal C2 domain and a C-terminal disordered region. The AlphaFold3-predicted BLTP-binding site in FAM102A and B is shown in J (see also Fig. S3 A). **(D)** Solo expression of FAM102A-GFP shows localization at the PM in U2OS cells, with no focal accumulations as expected for ER-PM contact sites. **(E)** In U2OS cells co-expressing FAM102A-GFP and BLTP2^Halo, the two proteins co-localize at ER-PM contacts. **(F)** In COS-7 cells, where BLTP2 does not accumulate at ER-PM contacts when expressed alone, co-expression of FAM102A-GFP recruits BLTP2^Halo to ER-PM contacts. **(G)** Solo expression of FAM102B-GFP in U2OS cells results in its diffuse localization. **(H)** In U2OS cells co-expressing FAM102B-GFP and BLTP2^Halo, the two proteins co-localize at ER-PM contacts. **(I)** In COS-7 cells, where BLTP2 does not accumulate at ER-PM contacts when expressed alone, co-expression of FAM102B-GFP recruits BLTP2^Halo to ER-PM contacts. **(J)** AlphaFold3 predicts an interaction between the C-terminal region of FAM102A (magenta) and a two-helix hairpin (green) (Fam102-binding motif, FBM) projecting out of the BLTP2 rod-like core (blue). **(K)** Left: COS-7 cells co-expressing BLTP2^EGFP and BLTP2^Halo-△FBM with FAM102A-iRFP showing that BLTP2^Halo-△FBM is not co-enriched with WT BLTP2^EGFP at ER-PM contact sites but remains diffuse throughout the ER. Right: Intensity profile of the red dashed line in the EGFP and Halo channels of the images at left shows the enrichment of BLTP2^EGFP (green) over BLTP2^Halo-△FBM (red) at PM patches. **(L)** The enrichment ratio of BLTP2^EGFP and BLTP2^Halo-△FBM at the basal surface when co-expressed with FAM102A-iRFP or FAM102B-iRFP are quantified. *n* = 15 cells for co-expressing FAM102A-iRFP and *n* = 9 cells for co-expressing FAM102B-iRFP. Mean ± SEM. **(M)** Top: Diagram of the FAM102B-EGFP and the SSPB-FAM102B-IDR-EGFP constructs. Bottom: Schematic drawing of the improved light-induced dimerization system to recruit FAM102B-IDR to the lysosomal membrane. **(N)** COS-7 cells expressing LAMP1-mCh-iLID show recruitment of SSPB-FAM102B-IDR-EGFP to LAMP1-positive organelles upon blue light activation, with only BLTP2-FL being co-recruited, but not BLTP2-ΔFBM.

**Figure S3.**
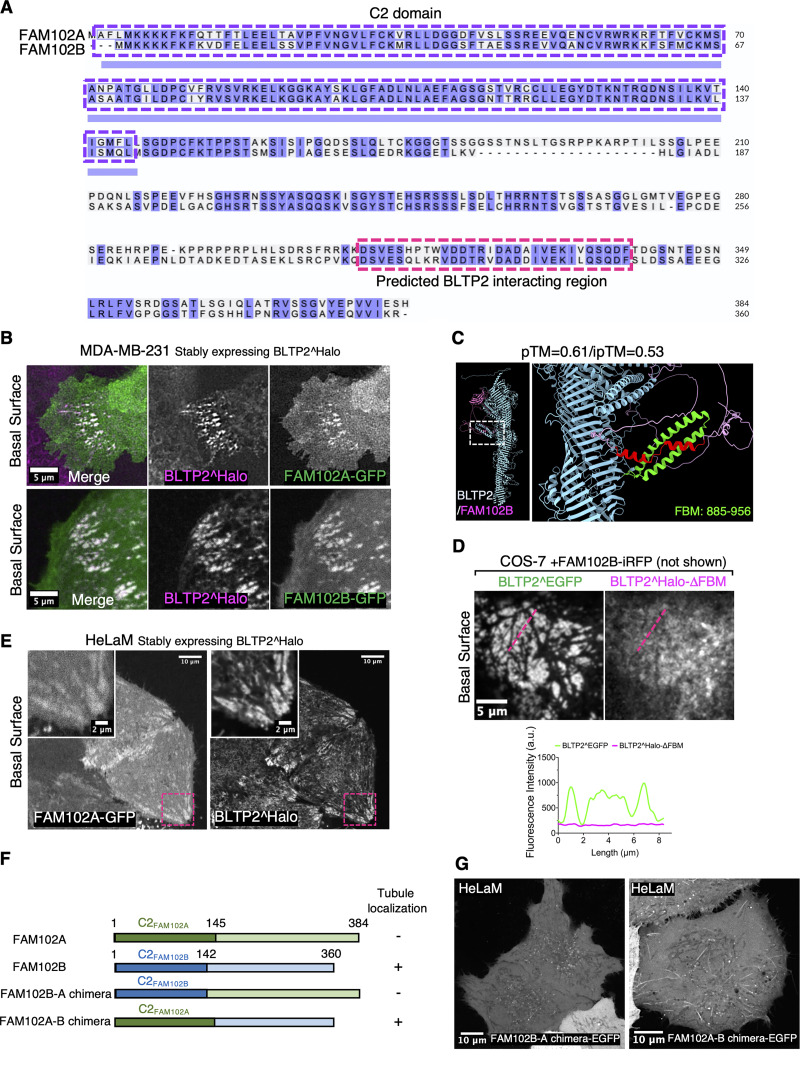
**Both FAM102A and FAM102B interact with BLTP2. (A)** Sequence alignment of human FAM102A and FAM102B, where their N-terminal C2 domains (purple dashed box) and their C-terminal predicted BLTP2-interacting regions (red dashed box) are highlighted. **(B)** Co-localization of FAM102A-GFP (top) and FAM102B-GFP (bottom) with BLTP2^Halo at ER-PM contact sites in MDA-MB-231 cells. **(C)** AlphaFold3 predicted interaction between FAM102B and BLTP2. **(D)** COS-7 cell co-expressing BLTP2^EGFP and BLTP2^Halo-△FBM with FAM102B-iRFP showing that BLTP2^Halo-△FBM is not co-enriched with WT BLTP2^EGFP at ER-PM contact sites but remains diffuse throughout the ER. **(E)** FAM102A-GFP recruits BLTP2^Halo to ER-PM contact sites in HeLaM cells. **(F)** Domain cartoon of the FAM102A-B chimeras. “+” indicates positive localization on tubular structures, and “−” indicates negative localization on tubular structures. **(G)** The FAM102A-B Chimera-EGFP is enriched at BLTP2-positive tubular structures in HeLaM cells.

A recently posted database of the yeast interactome (Michaelis et al., 2023) (http://yeast-interactome.biochem.mpg.de:3838/interactome/) revealed that the two yeast orthologs of BLTP2, Fmp27 and Ypr117w, are interactors of Ybl086c, with Fmp27 being the strongest interactor (Fig. 4, A and B). Interestingly, other interactors of Fmp27 are evolutionarily conserved proteins also implicated in lipid dynamics at ER-PM contacts (Ist2 [Wong et al., 2021] and Osh3 [Encinar Del Dedo et al., 2017]) or metabolism (Lro1 [Barbosa et al., 2019]). Ybl086c, in turn, has two orthologs in humans, FAM102A and FAM102B, which are similar proteins comprising an N-terminal C2 domain and a C-terminal intrinsically disordered region (IDR) (Fig. 4 C and Fig. S3 A). FAM102A, also named early estrogen-induced gene 1 (EEIG1), was identified as a receptor activator of nuclear factor κB ligand involved in the regulation of osteoclast formation and bone remodeling (Choi et al., 2013; Jeong et al., 2020; Wang et al., 2004). However, the cellular functions of both FAM102A and FAM102B are not known. As C2 domains can function as bilayer binding modules, we explored the possibility that these proteins could function as potential membrane adaptors for BLTP2 in mammalian cells.

First, we examined their localization when expressed as C-terminally tagged fusion proteins in U2OS cells or COS-7 cells. When expressed alone, both FAM102A-GFP and FAM102B-GFP had a diffuse PM and cytosolic localization (Fig. 4, D and G). However, when co-expressed with exogenous (and thus overexpressed) BLTP2^Halo, both FAM102A and FAM102B co-localized with BLTP2 at PM patches with the typical morphology of ER-PM contacts not only in U2OS cells (Fig. 4, E and H) and MDA-MB-231 cells (Fig. S3 B), but also in COS-7 cells (Fig. 4, F and I), where BLTP2^Halo, as mentioned above, does not exhibit an obvious concentration at ER-PM contacts (Fig. S1 B). These results suggest that FAM102A/B may help recruit BLTP2 to the ER-PM contacts, but they may be present in limiting concentration in COS-7 cells.

We then used AlphaFold3 to determine whether the FAM102 proteins may directly bind BLTP2 and found a high-confidence (pTM = 0.61/ipTM = 0.51) predicted interaction between a conserved sequence in the C-terminal region of FAM102A/B and an alpha-helix hairpin (hence called FBM for FAM-binding motif) that projects out from the rod core of BLTP2 (Fig. 4 J and Fig. S3 C). Supporting the physiological importance of this interface, a BLTP2 deletion construct lacking this motif (BLTP2^Halo-△FBM) was no longer recruited to PM patches by co-expression with either FAM102A or FAM102B in COS-7 cells, in contrast to full-length (FL) BLTP2^EGFP (Fig. 4, K and L; and Fig. S3 D).

To further confirm an interaction between BLTP2 and FAM102 proteins mediated by the FBM, we used the iLID-dependent heterodimerization system (Guntas et al., 2015). We generated a construct in which the C2 domain of FAM102B was replaced with the SSPB component of the system (SSPB-FAM102B-IDR-EGFP) and expressed it together with either FL BLTP2^Halo or the BLTP2^Halo-ΔFBM mutant (ΔFBM) in COS-7 cells, where a LAMP1 construct fused to the other components of the iLID system (LAMP1-mCh-iLID) was also expressed (Fig. 4 M). Before blue light illumination, SSPB-FAM102B-IDR-EGFP had a diffuse distribution in the cell, but after illumination it was quickly recruited to the LAMP1-positive organelles along with BLTP2-FL but not with BLTP2-ΔFBM (Fig. 4 N), validating the FBM-dependent interaction between BLTP2 and FAM102.

### The C2 domains of FAM102A and FAM102B bind PI(4,5)P_2_ at the PM

**Figure 5.**
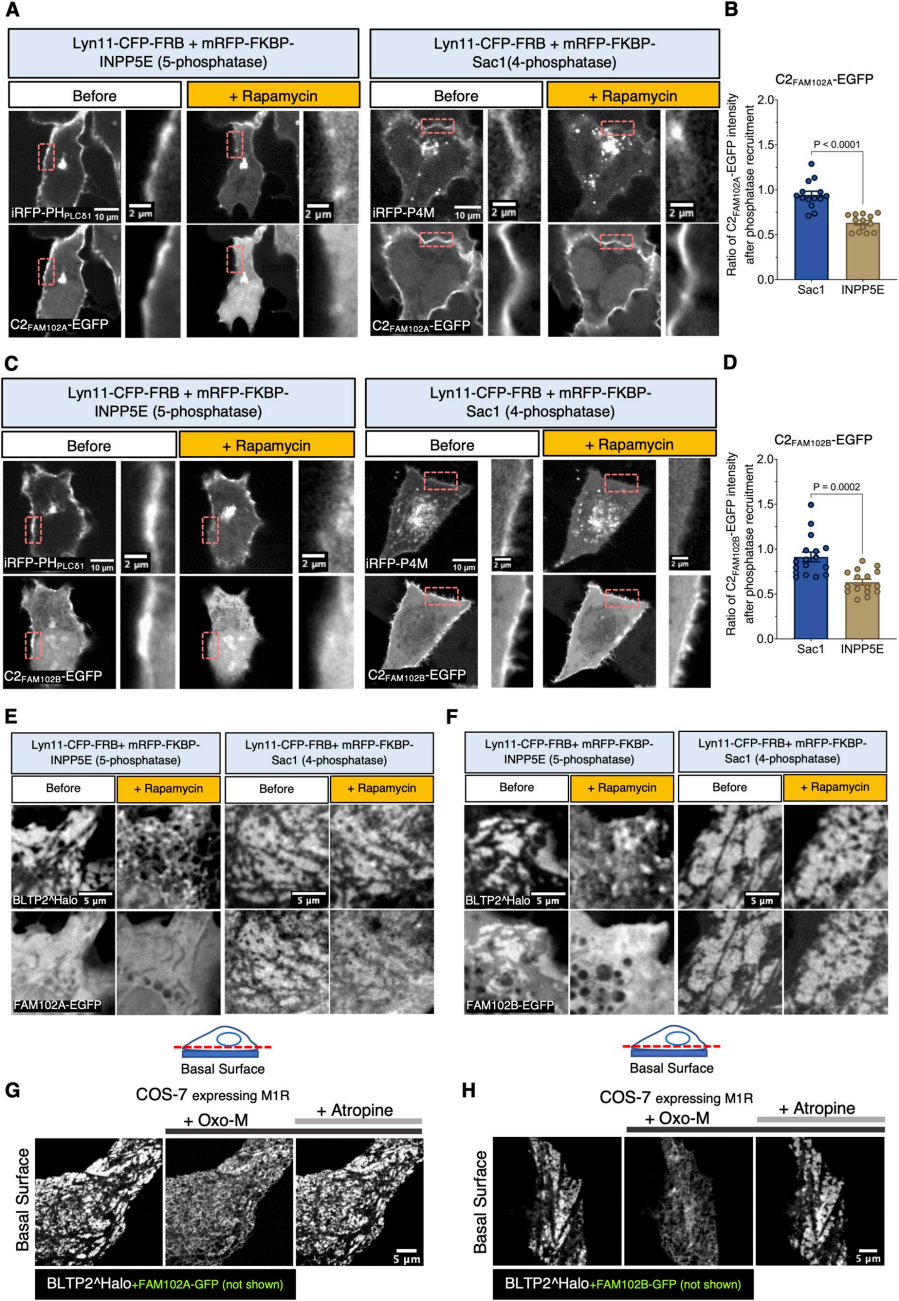
**FAM102A and FAM102B bind PI(4,5)P**_**2**_ **at the PM through their C2 domain. (A and B)** C2_FAM102A_-EGFP disassociates from the PM after the recruitment of a 5-phosphatase (INPP5E) but not after the recruitment of a 4-phosphatase (Sac1). The PI(4,5)P_2_ probe iRFP-PH_PLCδ1_ and the PI4P probe iRFP-P4M were used as positive controls to indicate the successful depletion of each phosphoinositide. The peak fluorescence intensity of C2 _FAM102A_ -EGFP on the PM was measured both before and after phosphatase recruitment and quantified as a ratio, as shown in B. Unpaired *t* test. *n* = 14 cells for Sac1 and *n* = 13 cells for INPP5E from three independent experiments. Mean ± SEM. **(C and D)** C2 _FAM102B_-EGFP was tested as in A and quantified as in D. Unpaired *t* test. *n* = 17 cells for Sac1 and *n* = 16 cells for INPP5E from three independent experiments. Mean ± SEM. **(E and F)** U2OS cells stably expressing BLTP2^Halo were transfected with either FAM102A-EGFP (E) or FAM102B-EGFP (F). The focal plane was adjusted to optimize visualization of ER-PM contacts at the basal surface. In both FAM102A and FAM102B co-expressing cells, BLTP2^Halo disassociated from the PM after PI(4,5)P_2_ but not after PI4P depletion. **(G and H)** In COS-7 cells co-expressing BLTP2^Halo, the muscarinic M1R receptor, and either FAM102A-GFP (G) or FAM102B-GFP (H), BLTP2^Halo disassociates from ER-PM contact sites and redistributes to the entire ER in response to PI(4,5)P_2_ depletion by Oxo-M addition. BLTP2^Halo relocalizes to ER-PM contacts after the addition of the Oxo-M antagonist atropine.

Many C2 domains bind the PM via an interaction with phosphoinositides (Cho and Stahelin, 2006). To assess a role of phosphoinositide binding in the recruitment of FAM102A and FAM102B to the PM via their C2 domains, we applied the rapamycin-induced dimerization system as described earlier to acutely manipulate phosphoinositides in the PM. When only the C2 domains of FAM102A and FAM102B were expressed in HeLa cells, they became enriched at the outer PM (with a more robust enrichment of C2_FAM102A_) (Fig. 5, A and C). Rapamycin-dependent acute recruitment of mRFP-FKBP-INPP5E to this membrane to deplete PI(4,5)P_2_ induced a dissociation of both C2 domains from the PM. However, acute recruitment of mRFP-FKBP-Sac1 to deplete PI4P had no effect (Fig. 5, A–D), demonstrating a specific role of P(I4,5)P2 in the binding to the PM of both C2 domains. Likewise, when the same rapamycin-induced dimerization system was used to examine the PI(4,5)P_2_ or PI4P dependence of BLTP2-FAM102A/B-positive contacts with the PM of U2OS cells, BLTP2, along with FAM102A/B, disassociated from the PM contacts upon PI(4,5)P_2_ depletion, but not upon PI4P depletion (Fig. 5, E and F). Dissociation of BLTP2^Halo from the PM in cells also expressing FAM102A-GFP or FAM102B-GFP was also observed when PI(4,5)P_2_ was acutely depleted through phospholipase C activation driven by the addition of Oxo-M to COS-7 cells expressing the muscarinic receptor M1R (Willars et al., 1998). These contacts were re-established after adding the muscarinic receptor antagonist atropine (Fig. 5, G and H; and Videos 3 and 4). Together, these results demonstrate a role of the binding of PM PI(4,5)P_2_ to the C2 domains of FAM102A and FAM102B in the formation of BLTP2-positive ER-PM contacts.

**Video 3.** **COS-7 cell co-expressing BLTP2^Halo (white), FAM102A-GFP (not shown), and M1R-blank (not shown) showing disruption of BLTP2^Halo-dependent ER-PM contacts after the addition of Oxo-M, with the dispersion of BLTP2^Halo throughout the ER and the re-establishment of such contacts after the addition of atropine.** 7 s intervals at 30 frames/s.

**Video 4.** **COS-7 cell co-expressing BLTP2^Halo (white), FAM102B-GFP (not shown), and M1R-blank (not shown) showing disruption of BLTP2^Halo-dependent ER-PM contacts after the addition of Oxo-M, with the dispersion of BLTP2^Halo throughout the ER and the re-establishment of such contacts after the addition of atropine.** 7 s intervals at 30 frames/s.

### FAM102B, but not FAM102A, is localized at PM-connected tubular structures in HeLaM cells

**Figure 6.**
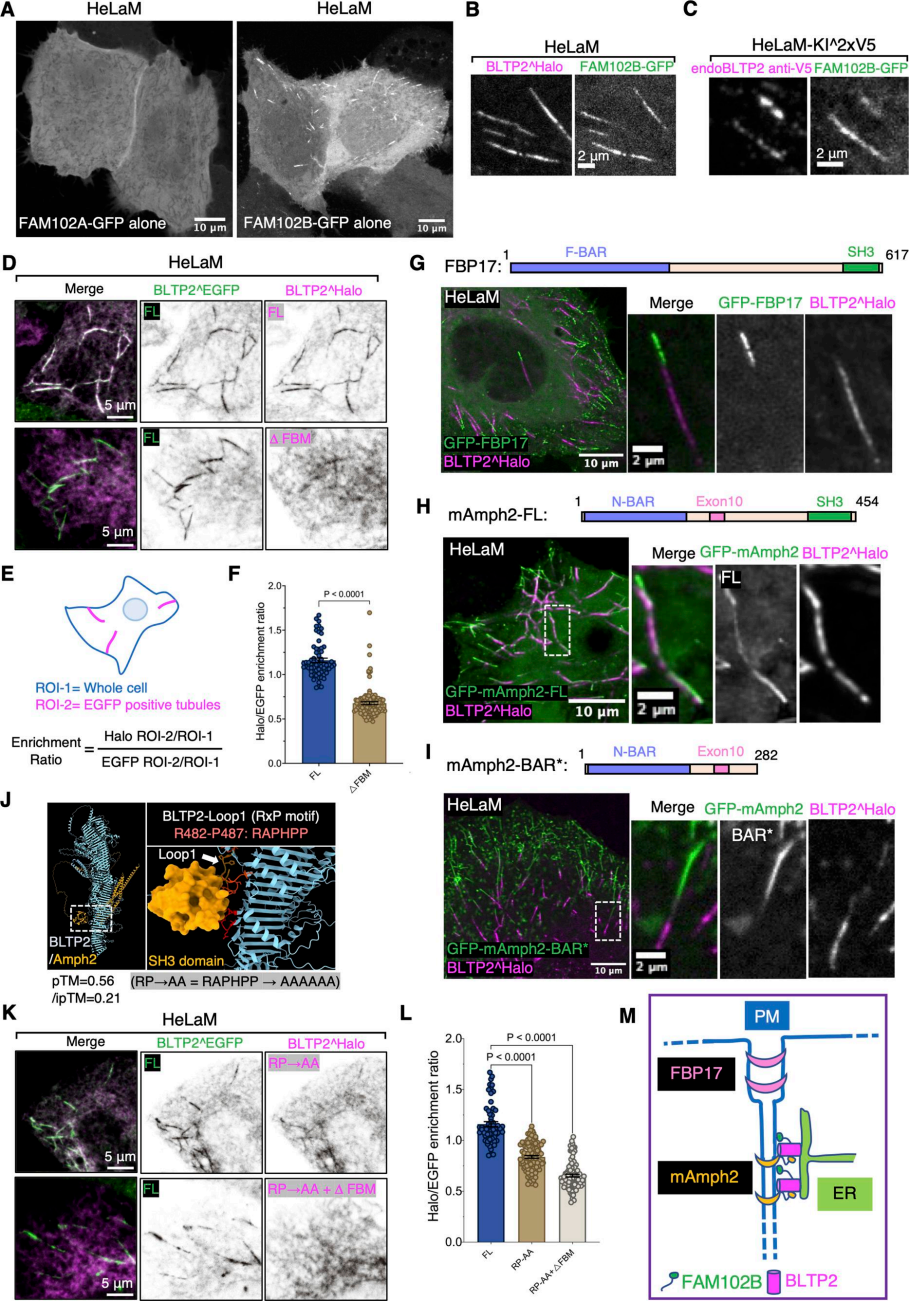
**Interactions of BLTP2 with FAM102B and N-BAR domain proteins at PM-connected tubular endosomes in HeLaM cells. (A)** Solo expression of FAM102A-GFP in HeLaM cells results in its diffuse localization, while solo expression of FAM102B-GFP results in its enriched localization on tubular structures similar to BLTP2 localization. **(B and C)** FAM102B-GFP co-localizes with either BLTP2^Halo (B) or endogenous BLTP2 (C) on tubular structures in HeLaM cells. **(D–F)** Co-expression of BLTP2^EGFP FL with either BLTP2^Halo FL or BLTP2^Halo-△FBM, respectively, in HeLaM cells, showing that deletion of the FBM motif decreases the enrichment of BLTP2 at the tubular structures (D). **(E)** shows the method used for the quantification. The quantification result is shown in F. Two-tailed *t* test. Mean ± SEM. (FL) *n* = 63 tubules from nine cells; (△FBM) *n* = 111 tubules from 15 cells. **(G)** GFP-FBP17 localizes on the same BLTP2^Halo-positive tubules but does not overlap with the BLTP2^Halo signal. **(H and I)** GFP-mAmph2 co-localizes with BLTP2^Halo on the tubular structures (H), while GFP-mAmph2-BAR* (I) localizes on the same tubules but does not overlap with BLTP2^Halo. **(J)** AlphaFold3 predicts the interaction of a loop of BLTP2 (loop1) with the SH3 domain of amphiphysin. This loop harbors a RAPHPP sequence (RxP motif), which fits an SH3 domain–binding consensus. **(K and L)** Co-expression of BLTP2^EGFP FL with a BLTP2^Halo construct in which each of these six aa of the SH3-binding consensus was mutated to alanine (BLTP2^Halo^RP→AA^), or BLTP2^Halo^RP→AA^ with the additional deletion of the FBM (BLTP2^Halo^RP→AA^-△FBM), showing a synergistic effect of abolishing SH3 domain binding and FAM102 binding in reducing the targeting of BLTP2 to the tubules. The quantification result is shown in L. One-way ANOVA. Mean ± SEM. (FL) *n* = 63 tubules from nine cells; (RP→AA) *n* = 103 tubules from 13 cells; (RP→AA and △FBM) *n* = 97 tubules from 13 cells. **(M)** Schematic drawing depicting co-localization of BLTP2, FAM102B, and amphiphysin 2 on the PM-connected tubular endosomes, but segregation form FBP17.

Surprisingly, when we expressed FAM102A and FAM102B in HeLaM cells, we observed that while FAM102A had a diffuse localization at the PM (Fig. 6 A), FAM102B was selectively enriched on the tubular structures (Fig. 6 A), where it precisely co-localized with both exogenous and endogenous BLTP2 (Fig. 6, B and C). However, when BLTP2^Halo was co-expressed with FAM102A-GFP, the two proteins co-localized at typical ER-PM patches on the basal surface of the cell (Fig. S3 E), suggesting that the selective localization of BLTP2 on the tubule in the absence of FAM102A overexpression reflects the predominant expression of FAM102B in these cells.

To determine whether the C2 domain of FAM102B is implicated in this specific property of FAM102B, we generated a chimera of FAM102B in which its C2 domain was replaced by the C2 domain of FAM102A (i.e., the FAM102 protein that does not localize to the tubules). We named it FAM102A-B chimera (Fig. S3 F). This chimera still localized to the tubules speaking against a role of the C2 domain in this localization (Fig. S3 G). The opposite chimera (FAM102B-A chimera) did not go to the tubules (Fig. S3 G). We conclude that it is the IDR of FAM102B that is required in its selective localization at tubules, possibly via indirect interactions, as the IDR does not contain obvious bilayer binding regions. When expressed alone, however, the IDR of FAM102B did not show any strong localization on any membranes (as shown before with the SSPB-FAM102B-IDR-EGFP in Fig. 4 N), indicating that the C2 domain may either contribute to bilayer binding of this region or release some autoinhibited configuration of the IDR.

### Interactions with endocytic Bin-amphiphysin-Rvs proteins cooperate with other factors in BLTP2 recruitment to PM-connected tubular endosomes

In our studies of HeLaM cells, we noticed that although the enrichment of BLTP2^Halo-△FBM relative to FL BLTP2^EGFP at surface-exposed tubules was strongly reduced, it was not completely abolished (Fig. 6, D–F). This implied that other factors may contribute to the enrichment of BLTP2 at the tubules. Tubular invaginations of the PM are sites where endocytic Bin-amphiphysin-Rvs (BAR) domain–containing proteins, for example, FBP17 and amphiphysin family proteins, are known to assemble via the curvature-sensing properties of their BAR domains (Frost et al., 2009; Itoh et al., 2005; Peter et al., 2004; Takei et al., 1999). Moreover, BAR domain–containing proteins were implicated in the biogenesis of tubular recycling endosomes (Giridharan et al., 2013; Gopaldass et al., 2024; Lucken-Ardjomande Häsler et al., 2020; Pant et al., 2009) and were observed at Rab10-positive PM invaginations (Zhu et al., 2024). Typically, these proteins function as adaptors to recruit to curved bilayers, often via SH3 domains, a variety of other factors, such as cytoskeletal scaffolds and signaling proteins (Itoh and De Camilli, 2006; Itoh et al., 2005; Tsujita et al., 2006). Thus, we explored whether BAR proteins could contribute to the recruitment of BLTP2 at the distal portion of tubular recycling endosomes.

**Figure S4.**
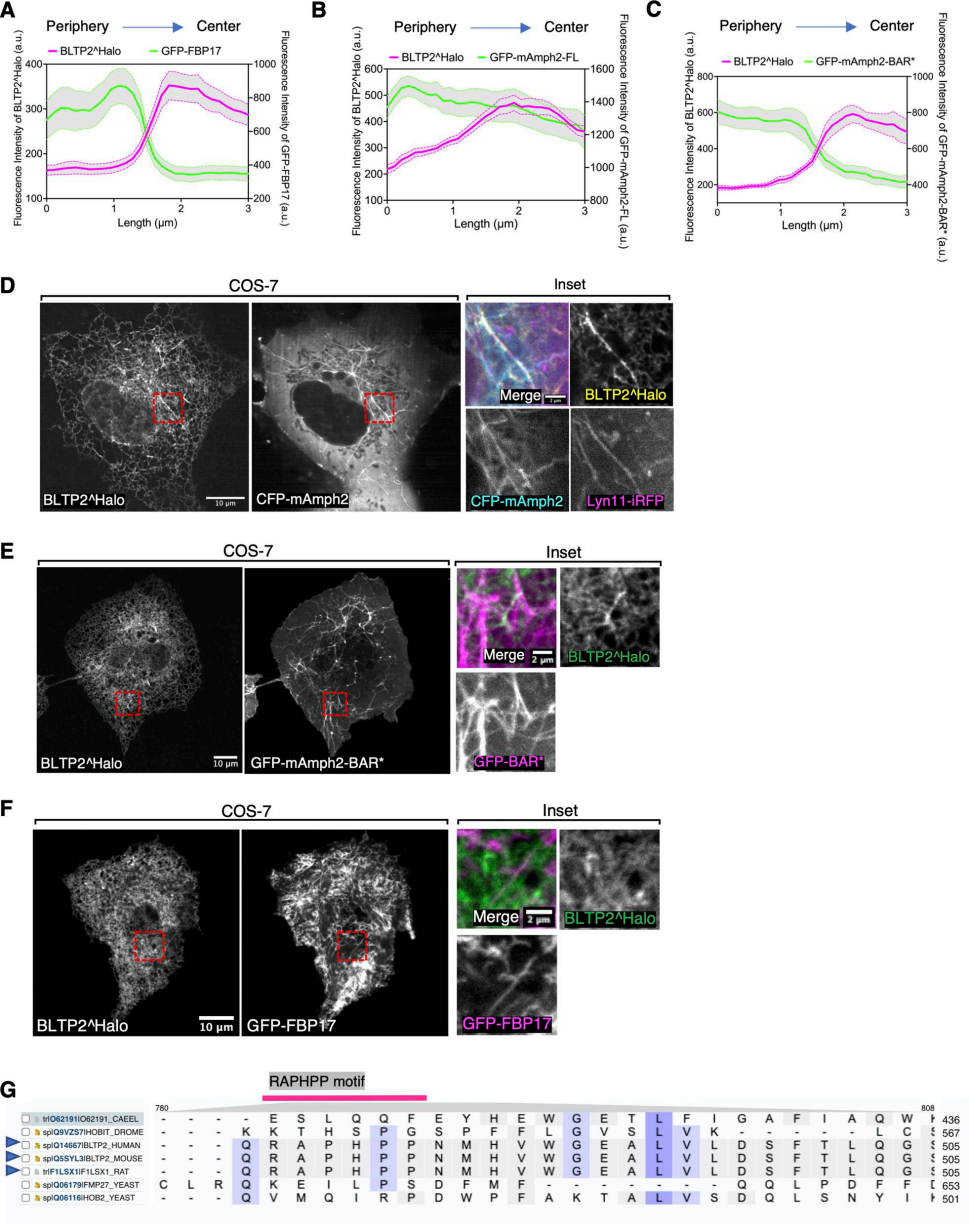
**BLTP2 interacts with amphiphysin 2 in an SH3 domain–dependent manner. (A–C)** A 3 μm line was drawn onto BLTP2-positive tubules in a peripheral (portion positive for BAR protein only) to center direction. The intensity profile of both BLTP2^Halo and the BAR domain proteins are shown. *n* = 25 tubules for each experiment. Corresponding to Fig. 6, G–I. Mean ± SEM. **(D)** COS-7 cells showing that BLTP2^Halo is recruited to PM tubular invaginations induced by expression of CFP-mAmph2. **(E)** COS-7 cells showing that BLTP2^Halo is not recruited to PM tubular invaginations induced by expression of GFP-mAmph2 BAR*, which lacks the SH3 domain. **(F)** COS-7 cells showing that BLTP2^Halo is not recruited to PM tubular invaginations induced by expression of GFP-FBP17. **(G)** Sequence alignment of the region comprising the RAPHPP motif among BLTP2 from several organisms. The RAPHPP motif is conserved among mammalian species (blue arrowhead).

When we expressed the F-BAR domain proteins FBP17 (GFP-FBP17) and the N-BAR domain protein amphiphysin 2 (GFP-mAmph2) (more precisely its non-neuronal isoform also referred to as BIN1) in HeLaM cells, both proteins, which contain C-terminal SH3 domains, became highly enriched on the BLTP2-positive tubular structures (Fig. 6, G and H). In agreement with previous studies of endocytic invaginations, GFP-FBP17, whose F-BAR domain has lower curvature than N-BAR domains, marked selectively the portion of the tubules closer to the outer PM (Frost et al., 2008; Wu et al., 2010) and was adjacent to, but did not overlap with, the BLTP2^Halo fluorescence (Fig. 6 G and Fig. S4 A). In contrast, GFP-mAmph2, which associates with smaller diameter tubules (Frost et al., 2008; Takei et al., 1999), was also localized more deeply into the tubules, where it precisely co-localized with BLTP2 (Fig. 6 H and Fig. S4 B). However, a construct of amphiphysin 2 lacking the SH3 domain (GFP-mAmph2-BAR*) still localized on the tubules but no longer co-localized with BLTP2 (Fig. 6 I and Fig. S4 C), confirming an SH3 domain–mediated interaction between the two proteins but also demonstrating that this interaction is not essential for the recruitment of BLTP2 to the tubules.

An interaction between BLTP2 and amphiphysin 2 was supported by overexpressing CFP-mAmph2 or GFP-Amph2-BAR* along with BLTP2^Halo in COS-7 cells that typically contain only a few tubular recycling endosomes. Both overexpressed Amph2 and Amph2-BAR* induced PM tubular invaginations via their curvature-generating properties, as expected (Lee et al., 2002). While the tubules of this artificial system are very different in their origin and properties from tubular recycling endosomes, BLTP2 was recruited to them when they were generated by Amph2, but not by Amph2-BAR* (Fig. S4, D and E), supporting an SH3-mediated interaction. Consistent with the results in HeLaM cells, BLTP2^Halo was not recruited to tubules generated by GFP-FBP17 (Fig. S4 F).

In agreement with these results, AlphaFold3 predicted binding of the SH3 domain of mAmph2 to an aa loop of BLTP2 that projects out of its rod-like core near its N terminus (pTM = 0.55) (Fig. 6 J). This loop contains the core SH3 binding consensus “RxP” motif (Kurochkina and Guha, 2013) within the “RAPHPP” sequence, which is conserved among mammalian BLTP2s (Fig. S4 G). When a BLTP2 construct in which all these six aa had been mutated to alanine (BLTP2^Halo^RP→AA^) was expressed in HeLaM cells, its enrichment on the tubules relative to the enrichment of BLTP2^EGFP-FL was reduced (Fig. 6, K and L). Moreover, combining this mutation with the deletion of the FBM (BLTP2^Halo^RP→AA^-△FBM) almost completely abolished its enrichment on the tubules (Fig. 6, K and L). We conclude that both FAM102B and SH3 domain N-BAR domain proteins contribute to recruiting BLTP2 to PM-connected tubular endosomes (Fig. 6 M).

### BLTP2 is recruited to macropinosomes undergoing fusion with PM

In the course of our live-imaging experiments involving cells expressing fluorescently tagged BLTP2, we sometimes observed transient flashes of focal BLTP fluorescence close to the cell surface. When these experiments were performed in the presence of anti–MHC-I antibodies to label broadly the PM and macropinosomes, we found that these flashes correspond to the exocytosis of macropinosomes. This was clearly exemplified by experiments in COS-7 cells, which have only a few focal BLTP2^Halo accumulations at ER-PM contacts (unless co-expressed with FAM102) and therefore allow easy visualization of these events.

**Figure 7.**
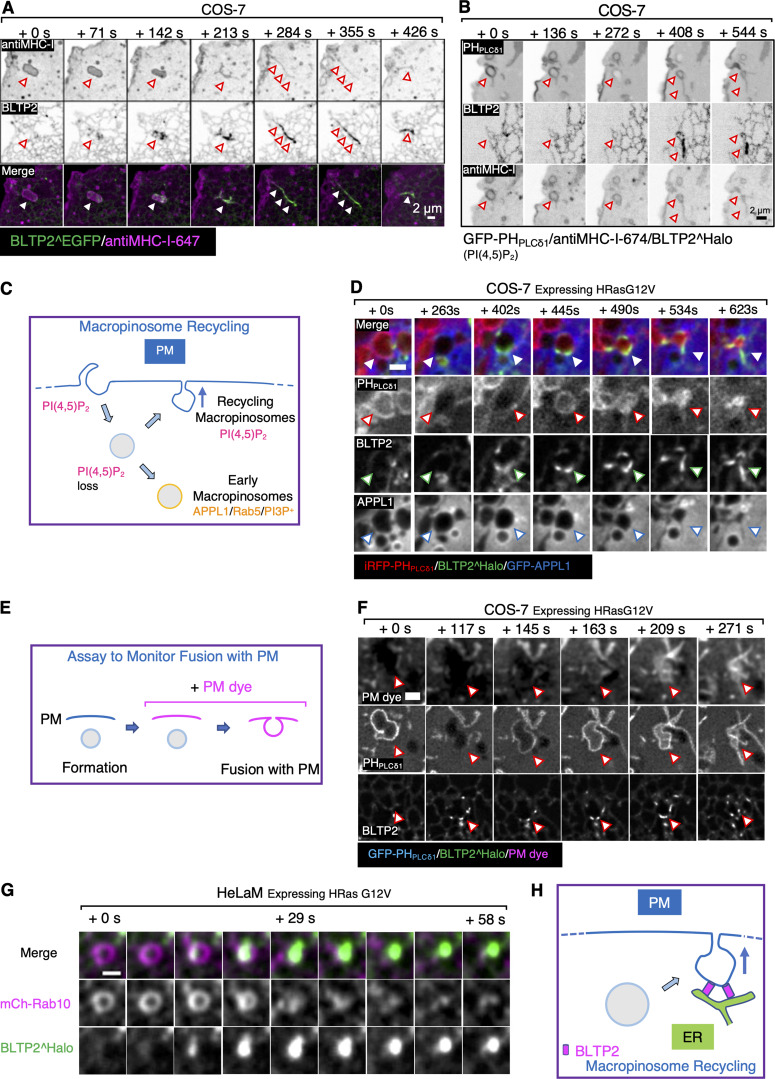
**BLTP2 is recruited to recycling macropinosomes undergoing fusion with the PM. (A and B)** COS-7 cells showing macropinosomes (labeled by internalized fluorescent anti–MHC-I antibodies) that undergo a dramatic morphological change and acquire BLTP2^EGFP as they fuse and collapse with the PM. In B, a newly formed macropinosome at first loses PI(4,5)P_2_, but then reacquires PI(4,5)P_2_ as it fuses with the PM. **(C)** Schematic drawing of macropinosome recycling. A nascent macropinosome first loses PI(4,5)P_2_ after fission from the PM, then it acquires the identity of early endosomes (PI3P, APPL, and Rab5) or regains PI(4,5)P_2_ as it fuses back to the PM. **(D)** Stimulation of macropinocytosis by expression of HRas G12V in COS-7 cells. BLTP2^Halo is recruited to a newly formed macropinosome that loses PI(4,5)P_2_ but does not acquire APPL2 and regains PI(4,5)P_2_ signaling during its recycling back to the PM. Scale bar, 2 μm. **(E)** Schematic drawing depicting the assay to monitor macropinosome fusion with the PM. PM dye is added after the formation of macropinosomes. Preformed macropinosomes will not be labeled by the dye until they fuse with the PM. **(F)** A BLTP2-positive macropinosome gains access to the PM dye, showing that it fuses with the PM. Macropinocytosis was stimulated by expressing HRas G12V. Scale bar, 2 μm. **(G)** Acute recruitment of BLTP2^Halo to a macropinosome in the process of fusing with the PM in HeLaM cells. The macropinosome is also positive for mCherry-Rab10. Scale bar, 1 μm. **(H)** Schematic drawing of the recruitment of BLTP2 to a recycling macropinosome undergoing fusion with the PM. Scale bar, 1 μm.

**Figure S5.**
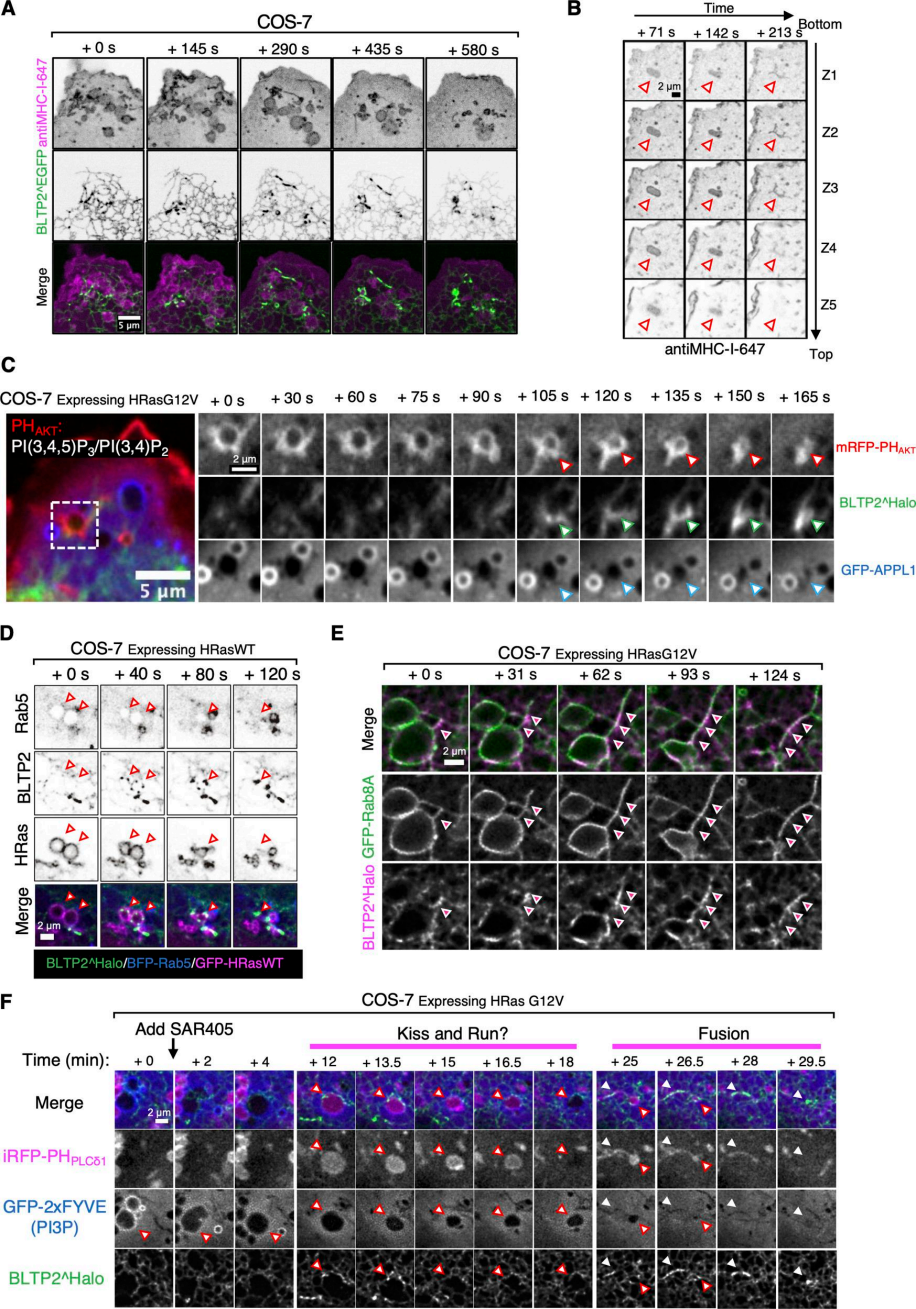
**BLTP2 is recruited to macropinosomes undergoing fusion with the PM in COS-7 cells. (A)** Acute recruitment of BLTP2^EGFP to macropinosomes (labeled by internalized fluorescent anti–MHC-I antibodies) in the process of fusing with the PM. **(B)** Z-stack of the time-lapse images shown in Fig. 7 A, proving that the macropinosome is not moving out from the focal plane during the imaging session. **(C)** Recycling macropinosomes that acquire BLTP2 signal remain positive for PH_AKT_ and do not transition to the APPL1 stage. **(D)** BLTP2-positive recycling macropinosomes are not positive for Rab5. **(E)** BLTP2-positive recycling macropinosomes are positive for the Rab8A, an exocytic Rab. **(F)** Upon treatment of cells with the VPS34 inhibitor SAR405, the PI3P-positive early macropinosomes can reacquire PI(4,5)P_2_ and recycle back to the PM.

Most macropinosomes, shortly after internalization, acquired as expected markers of early endosomal stations, such as Rab5, its effector APPL1 and PI3P (Buckley and King, 2017; Donaldson, 2019; Zoncu et al., 2009). However, a subset of them did not progress to this stage and instead acquired bright transient spots of BLTP2 signals, revealing the formation at their surface of contacts with the ER where BLTP2 is highly concentrated. Strikingly, the appearance of BLTP2 on these macropinosomes correlated with a major change of their shape. They shrank and transformed from vacuolar structures into tubules that appeared to be connected to the PM while remaining BLTP2 positive. Eventually these structures disappeared, suggesting their collapse into the PM, with a corresponding loss of the BLTP2 signal, as confirmed by Z-stacks, which ruled out their moving out of the focal plane (Fig. 7 A; Fig. S5, A and B; and Videos 5 and 6).

**Video 5.** **COS-7 cell showing a macropinosome labeled by anti–MHC-I-647 antibodies that undergoes a drastic morphological change as it fuses with the PM.** This transition correlates with the formation of BLTP2^EGFP-positive ER contacts. 7 s intervals at 60 frames/s.

**Video 6.** **COS-7 cell showing multiple newly formed macropinosomes labeled by anti–MHC-I-647 antibodies that undergo drastic morphological change as they fuse with the PM and acquire patches of BLTP2^EGFP during this transition.** 7 s intervals at 60 frames/s.

The fusion of these organelles with the PM was confirmed by monitoring the dynamics of PI(4,5)P_2_, a defining lipid of the PM (Di Paolo and De Camilli, 2006), on their surface. In cells expressing the PI(4,5)P_2_ probe PH_PLCδ1_, PI(4,5)P_2_ disappeared from nascent macropinosomes as expected, as PI(4,5)P_2_ is known to be rapidly removed from endocytic vesicles, primarily via the action of PI(4,5)P_2_ phosphatases (Maxson et al., 2021). However, on the macropinosomes that had acquired BLTP2 signal, a concomitant resurgence of PI(4,5)P_2_ was observed (Fig. 7 B and Video 7), as expected if they had fused with the PM and thus could acquire this phospholipid via its diffusion from the surrounding PM bilayer. Most likely, acquisition of PI(4,5)P_2_ is a key signal that triggers formation of BLTP2-dependent ER-PM tethers on the macropinosomes that undergo exocytosis.

**Video 7.** **COS-7 cell showing a newly formed macropinosome labeled by anti–MHC-I-647 antibodies that first loses PI(4,5)P**_**2**_ **(as detected by GFP-PH**_**PLCδ1**_ **) just after internalization, then reacquires PI(4,5)P**_**2**_ **as it fuses with the PM.** The acquisition of PI(4,5)P_2_ (which signals fusion with the PI(4,5)P_2_-rich PM) correlates with the formation of BLTP2^Halo-positive ER contacts. 9 s intervals at 60 frames/s.

Similar results were obtained in COS-7 and HeLaM cells, where macropinocytosis was induced by the expression of the constitutively active mutant form of HRas (HRas G12V) (Porat-Shliom et al., 2008). Even in these cells we found that after losing PI(4,5)P_2_, some macropinosomes, which were labeled by the PI(3,4,5)P_3_/PI(3,4)P_2_ marker PH_AKT_ as expected (Bohdanowicz and Grinstein, 2013; Levin et al., 2015; Swanson and Araki, 2022), did not mature into the APPL1 or Rab5 stage (Fig. 7 D; and Fig. S5, C and D). Instead they retained the PI(3,4,5)P_3_/PI(3,4)P_2_ signal and then showed BLTP2 recruitment correlated with shrinking (Fig. S7 C). To confirm that PI(4,5)P_2_ resurgence reflected fusion events with the PM, we added to cells the non-permeable membrane dye CellBrite steady 650 that cannot have access to macropinosomes generated before its addition (Fig. 7 E). Observation of these cells showed that resurgence of PI(4,5)P_2_ on macropinosomes coincided with their labeling by CellBrite steady 650, thus indicating opening of their lumen to the cell surface (Fig. 7 F and Video 8). Finally, macropinosomes that became positive for PI(4,5)P_2_ and BLTP2 were also positive for Rab8 and Rab10, two Rab GTPases implicated in exocytosis of a variety of vesicles (Das and Guo, 2011; Larance et al., 2005; Sano et al., 2007; Stenmark, 2009), including macropinosomes (Spangenberg et al., 2021, *Preprint*) (Fig. 7 G and Fig. S5 E).

**Video 8.** **COS-7 cell expressing HRas G12V and showing a newly formed PI(4,5)P**_**2**_ **-positive (GFP-PH**_**PLCδ1**_ **signal) macropinosome that loses PI(4,5)P**_**2**_ **, but then reacquires it along with the PM dye signal as it fuses back to the PM. **BLTP2^Halo is also recruited during its fusion with the PM. 8 s intervals at 60 frames/s.

The occurrence of macropinosomes that fail to acquire early endocytic markers, acquire instead Rab8 and Rab10, and recycle back to the PM is in agreement with other studies (Liu et al., 2020; Spangenberg et al., 2021, *Preprint*). It was also reported that the proportion of these recycling events is strongly increased upon inhibition of VPS34 (Spangenberg et al., 2021, *Preprint*), the kinase that generates on early endosomes PI3P, the signature phosphoinositide of these organelles. Accordingly, upon treatment of COS-7 cells with the VPS34 inhibitor SAR405 (Spangenberg et al., 2021, *Preprint*), we observed many PI3P-positive macropinosomes that reacquired PI(4,5)P_2_, became positive for BLTP2, and fused with the PM (Fig. S5 F). We conclude that BLTP2-positive ER contacts occur at macropinosomes that are undergoing fusion with the PM, suggesting a role of BLTP2 lipid transport function as the macropinosome membrane becomes part of the PM (Fig. 7 H).

### Absence of BLTP2 impairs the collapse of macropinosomes into the PM after their fusion

**Figure 8.**
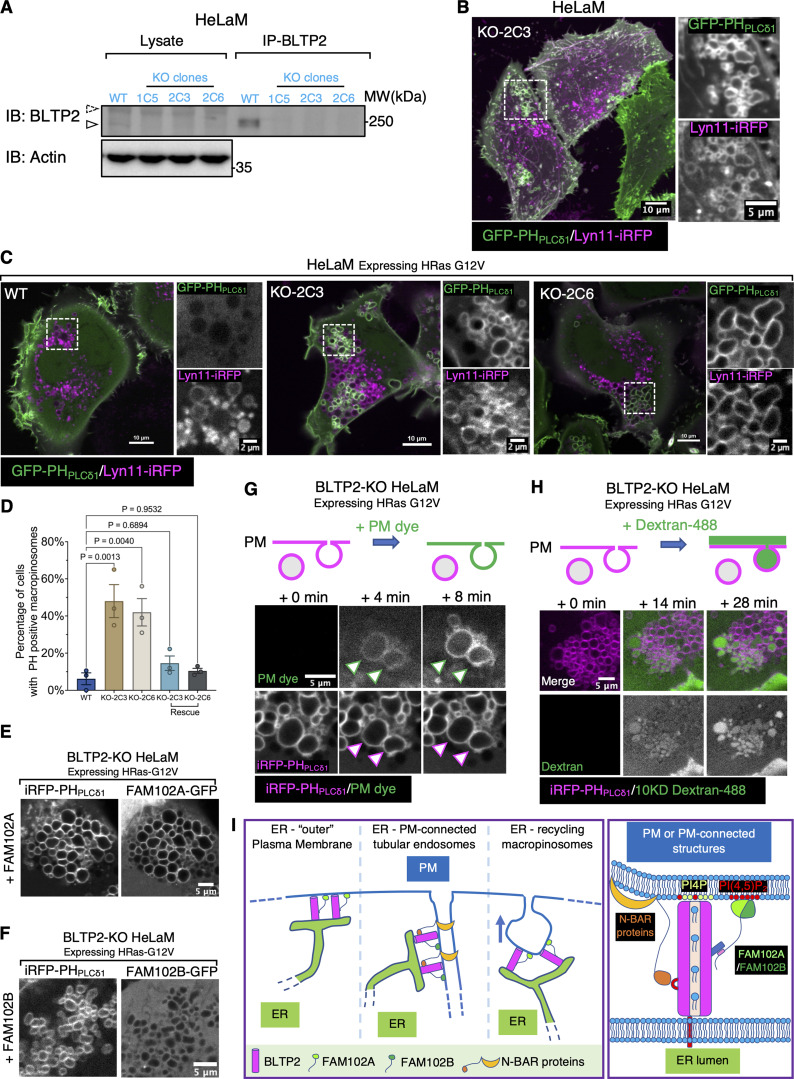
**BLTP2-KO cells show accumulation of intracellular PI(4,5)P**_**2**_ **-positive vacuoles. (A)** Western blot validating knockout (KO) of BLTP2 in HeLaM cells. Endogenous BLTP2 is enriched using IP before detection. Three independent KO clones are verified. **(B)** BLTP2-KO HeLaM cells showing presence of PI(4,5)P_2_-positive intracellular vacuoles. Tubular recycling endosomes are still present in these cells. **(C and D)** Expression of HRas G12V in HeLaM cells (two independent clones: KO-2C3 and KO-2C6) induces formation of macropinosomes/intracellular vacuoles in both WT and BLTP2-KO cells. However, only in the KO cells a large fraction of these vesicles remain PI(4,5)P_2_ positive. Quantification of PI(4,5)P_2_-positive macropinosomes is shown in D. One-way ANOVA. Mean ± SEM. *n* = 3 independent experiments. 123 cells for WT, 137 cells for KO-2C3, 161 cells for KO-2C6, 119 cells for KO-2C3 rescue, and 122 cells for KO-2C6 rescue. **(E and F)** BLTP2-KO cells expressing HRas G12V together with FAM102A-GFP (E) or FAM102B-GFP (F). FAM102A-GFP, but not FAM102B-GFP, is enriched on PH_PLCδ1_-labeled PI(4,5)P_2_-positive macropinosomes. **(G and H)** PM dye (G), or 10 KD dextran-488 (H) was added to BLTP2-KO cells expressing HRas G12V. A fraction of the preexisting PI(4,5)P_2_-positive vacuoles were labeled by the dye within minutes after PM dye addition (G), showing internalization of dextran-488 (H). **(I)** Schematic model of BLTP2 localization at contacts of the ER with PM and PM-connected structures (left). Illustration of the molecular interaction of BLTP2 with phosphoinositides and its binding proteins (FAM102A/B, N-BAR domain proteins) at these contact sites (right). Source data are available for this figure: SourceData F8.

Collectively, the results reported above suggest that a primary localization of BLTP2 is at contacts between the ER and the PM. Such localization, in turn, suggests that BLTP2-dependent transport of lipids from the ER to the PM may be required to define and maintain the properties of the PM, with an important impact on its dynamics. To address this possibility, we generated BLTP2-KO HeLaM cells using CRISPR-Cas9–based strategy. Single KO clones were isolated and verified both by nucleotide sequencing of the edited region and by western blotting (Fig. 8 A).

Inspection of WT and BLTP2 KO cells revealed formation of PI(4,5)P_2_-positive (as revealed by PH_PLCδ1_ labeling) vacuoles in the KO cells, while the PM-connected tubular endosomes seem undisrupted (Fig. 8 B). However, upon expression of constitutively active RAS (HRas G12V), not only did most KO cells show a massive accumulation of macropinosomes relative to controls, but also many of these macropinosomes, in contrast to those present in WT cells, were positive for PI(4,5)P_2_ (Fig. 8, C and D) and for FAM102A (Fig. 8 E), but not FAM102B (Fig. 8 F). Additionally, many of these PI(4,5)P_2-_ and FAM102A-positive vacuoles were connected to the PM, as they were accessible to both the non-permeable membrane dye CellBrite steady 650 (Fig. 8 G and Video 9) and to 10 kD dextran-488 (Fig. 8 H and Video 10). Importantly, this phenotype was rescued by exogenous expression of BLTP2^Halo (Fig. 8 D), confirming its BLTP2 dependence. Based on these observations, we suggest that these vacuoles represent postfusion structures whose collapse into the PM is impaired or macropinosomes that failed to undergo fission from the PM. Irrespective of the mechanisms underlying their formation, these results indicate that loss of BLTP2 has a major impact on the dynamics of the PM.

**Video 9.** **BLTP2-KO HeLaM cell expressing HRas G12V showing that a subset of the internally accumulated vacuoles positive for PI(4,5)P**_**2**_ **(iRFP-PH**_**PLCδ1**_ **) become labeled with the PM dye CellBrite shortly after the addition of this dye, signaling continuity of these vacuoles with the PM. **KO, knockout. 58 s intervals at 60 frames/s.

**Video 10.** **BLTP2-KO HeLaM cell expressing HRas G12V, showing that upon addition to the cells of 10 KD dextran-488, the lumen of a subset of the internally accumulated vacuoles becomes positive for this marker within a few minutes, signaling their accessibility to the extracellular medium.** KO, knockout. 30 s intervals at 60 frames/s.

## Discussion

Our study shows that BLTP2 is primarily concentrated at contacts between the ER and the PM in commonly used mammalian cell lines, although with cell-specific features, and identifies some of the interactions responsible for these localizations (Fig. 8 I). We also show that the absence of BLTP2 results in the presence of intracellular vacuoles with PM-like properties and, at least in some cases, still connected to the PM, consistent with a role of this protein in controlling the dynamics of the cell surface, most likely via its properties to transport phospholipids from the ER to the PM.

Our findings reconcile discrepancies from the previous literature, which had reported a localization of BLTP2 orthologs at ER-PM contacts in yeast, *Drosophila*, and mammalian cell lines (Banerjee et al., 2025; Neuman et al., 2022a), but a selective localization at contacts between the ER and tubular recycling endosomes in another study of mammalian cells (Parolek and Burd, 2024). We confirmed that the tubules reported by this study have the reported properties of tubular recycling endosomes, as they are positive for Rab8 and Rab10 as well as for proteins, such as MHC-I, found to populate such endosomes. However, we found that, when positive for BLTP2, these tubular membranes are connected to the PM and thus represent another example of ER-PM localization. The connection to the PM of Rab10-positive tubules was also recently reported in a study which describes these tubules as PM invagination (Zhu et al., 2024). These tubules are very abundant in HeLaM cells, where they are typically very long, often extending from the Golgi complex region to the PM. At least in most cases, these tubular structures do not appear to be typical transport intermediates, but lasting structures. Moreover, they appear to be heterogeneous in properties along their length, as they have *bona fide* PM properties selectively on their portions closer to the outer cell surface, as exemplified by the formation of BLTP2-dependent tethers and by the presence of other classical ER-PM tethers, such as TMEM24 and MAPPER, selectively in these regions. Similar tubules with an enrichment of contacts with the ER at their surface were described by Yao et al. (2018), *Preprint*. It is possible that a special concentration of BLTP2 and other lipid transport proteins at these sites may help ensuring PM-like lipid composition on the distal portions of the tubules, which are directly adjacent to, and continuous with, the outer plasma membrane.

Other sites where we have detected presence of BLTP2-positive ER contacts are macropinosomes in the process of fusing with the PM. These are macropinosomes that fail to migrate and mature to late stations in the endocytic pathway and instead recycle back to the PM. As they fuse with the PM, such macropinosomes undergo a dramatic shrinkage and remodeling of their membrane, which correlates with the recruitment of BLTP2-positive contacts. BLTP2-mediated lipid transport at this stage may help provide lipids for this remodeling or to make the membrane of macropinosome compatible with its intermixing with the PM. The appearance of flashes of BLTP2 fluorescence on macropinosomes (reflecting acute formation of BLTP2-positive ER-PM contacts) only when they have established continuity with the PM, provides a striking demonstration of the PM-binding specificity of BLTP2.

Concerning mechanisms through which ER-anchored BLTP2 binds *in trans* to the PM to achieve bridge-like lipid transport, we have found evidence for both lipid-based and protein-based interactions. We have shown that phosphoinositides in the PM are important. Moreover, we have identified FAM102A and FAM102B as PM-associated adaptor proteins that bind BLTP2 via an interaction conserved from yeast to mammals, as it was independently found that the yeast ortholog of FAM102A/B (Hoi1/Ybl086c) binds to, and targets, the yeast BLTP2 orthologs (Fmp27 and Ypr117w) to ER-PM contacts (Dziurdzik et al., 2025, *Preprint*). Clearly, the overexpression of these two proteins enhances BLTP2 targeting to the PM. Conversely, removal of the FAM102-binding motif from BLTP2 strongly decreases, although does not abolish, its PM targeting, indicating that FAM102 proteins are not the only determinants of BLTP2 localization. While these two proteins, which comprise an N-terminal C2 domain followed by an ∼240-aa long predicted unfolded region, are very similar to each other, they also have some different properties, as only FAM102B selectively accumulates with BLTP2 at the tubular invaginations of the PM, which are continuous with tubular recycling endosomes. Interestingly, the yeast interactome database, which reports a high-confidence interaction of the yeast BLTP2 ortholog Fmp27 with the yeast Fam102 protein (Ybl086c), also identifies Osh3, Ist2, and Lro1 as components of the Fmp27- Ybl086c network (Fig. 4 A). Both Ist2 (TMEM16 in mammals) and Osh3 (a member of the mammalian ORP family) are proteins implicated in lipid dynamics at ER-PM contacts (Encinar Del Dedo et al., 2017; Wong et al., 2021), while Lro1 (LCAT in mammals) is a phospholipid-metabolizing enzyme (Barbosa et al., 2019), raising the possibility of a functional cooperation with these proteins with BLTP2. Finally, we found evidence for SH3 domain–dependent interactions of BLTP2 with endocytic BAR domain proteins, such as amphiphysin 2, which may help better explain the concentration of BLTP2 on the tubules.

A recent study reported that BLTP2 and its orthologs in yeast play a role in controlling the appropriate fluidity of the PM bilayer by preferentially delivering phosphatidylethanolamine from ER (Banerjee et al., 2025). How BLTP2 could control a specific enrichment of this phospholipid in the PM remains unclear, but an impact of BLTP2 on PM fluidity could be one explanation for the abundant presence of intracellular vacuoles connected to the PM in BLTP2-KO cells, more so in cells expressing HRas G12V to stimulate bulk endocytosis. This accumulation may reflect impairment of the ability of this membrane to appropriately remodel in response to incoming and outgoing membrane traffic. Ectopic PI(4,5)P_2_ on intracellular membrane was also observed in *Drosophila* BLTP2 mutant cells (Neuman et al., 2022a), although the potential connection of these vacuoles to the PM was not explored. Another BLTP, BLTP1, which shares special similarities to BLTP2 (Levine, 2022; Toulmay et al., 2022), including the presence of a transmembrane region anchored to the ER, was also reported to control fluidity of PM, in this case by ensuring delivery of phospholipid with specific fatty acid composition (primarily saturation level of their aliphatic chains) (John Peter et al., 2022a; John Peter et al., 2022b; Toulmay et al., 2022; Wang et al., 2022). The importance of both BLTP1 and BLTP2 for organismal life, proven by early embryonic lethality of KO mice and by the developmental defects of BLTP1 and BLTP2 *Drosophila* mutants (Neuman and Bashirullah, 2018; Verstreken et al., 2009), are striking demonstrations that direct protein-mediated bulk lipid transport from the ER to the PM, a process unknown until recently, has a general and fundamental importance in cell physiology.

## Materials and methods

Key resources used in this study are listed in Table S1.

### Plasmids

The original clone containing the BLTP2 ORF (NCBI reference sequence: NM_014680.5) was obtained from GenScript. Internally tagged BLTP2^EGFP was generated by first amplifying the N-terminal (aa 1–724) and C-terminal (aa 725–2235) fragments of BLTP2 using PCR, respectively. The N-terminal fragment was inserted into a pEGFP-C1 plasmid between the NheI and AgeI restriction sites using the In-Fusion system (Takara) to create an intermediate construct “Nterm-1-724_EGFP”. The C-terminal fragment was then inserted in this intermediate construct between the XhoI and SalI restriction sites to create the full BLTP2^EGFP construct. For BLTP2^Halo, EGFP was replaced with a Halo tag. The full BLTP2^EGFP or BLTP2^Halo sequence was further amplified and inserted between the XbaI and EcoRI restriction sites in a pCAG vector using the In-Fusion system (Takara), respectively.

BLTP2 C-terminal truncation constructs were generated by amplifying each fragment and inserting them into the Nterm-1-724_EGFP construct. BLTP2^Halo-△FBM was generated using overlap PCR to remove the FBM sequence. The BLTP2^Halo^RP→AA^ and BLTP2^Halo^RP→AA^-△FBM mutation constructs were generated using over-lap PCR to replace the region to be mutated.

Both FAM102A (NCBI reference sequence: NM_001035254.3) and FAM102B (NCBI reference sequence: NM_001010883.3) cloned in a pcDNA3.1-C-eGFP vector between the KpnI and BamHI restriction sites were generated by GenScript. FAM102A-iRFP and FAM102B-iRFP were generated by swapping the EGFP with iRFP between the NotI and BsrGI restriction sites. The constructs encoding C2_FAM102A_-EGFP and C2_FAM102B_-EGFP were generated by synthesizing the nucleotide sequences of each C2 domain with a short C-terminal flanking sequence (C2_FAM102A_: aa 1–183, C2_FAM102B_: aa 1–180) (IDT) and inserting them into the pcDNA3.1-C-eGFP vector between the KpnI and BamHI restriction sites. For the SSPB-FAM102B-IDR-EGFP construct, a SSPB sequence was synthesized and inserted into the FAM102B-EGFP plasmid between the KpnI and BstEII sites (this digestion removed the first 142 aa of FAM102B). For the FAM102A-B chimeras, each chimera was synthesized by switching the C2 domain of each protein and inserting the resulting construct into the pcDNA3.1-C-eGFP vector between the KpnI and BamHI restriction sites.

mCherry-Rab10 T23N was generated using overlap PCR to introduce the point mutation. All primers and other constructs used in this study were listed in the key resource table.

### Cell culture and transfection

All cell lines were cultured at 37°C and 5% CO_2_ in DMEM supplemented with 10% FBS, 1× penicillin-streptomycin, and 1× GlutaMAX. For transient transfection, cells were first seeded in a 35-mm glass-bottom dish (MatTek). When cells reached 60–80% confluency, 1–3 μg plasmids and 2 μl Lipofectamine 2000 were diluted in pre-warmed Opti-MEM for 5 min, respectively, and then mixed for another 15 min before their addition to the cells.

### Generation of BLTP2 stable cell lines using lentivirus

BLTP2^Halo and BLTP2^EGFP were cloned into a pSIN vector, respectively. This vector was mixed with packaging vectors pMD2.G and pCMVR 8.74 and transfected into 293-T cells for virus production. 24 h after transfection, fresh medium was added to the cells. Medium containing the viruses was collected after another 24 h. Viruses were concentrated using an Lenti-X concentrator following the manufacture’s protocol by mixing 1× volume of the concentrator with 3× volume of clarified medium (by passing through a 0.45-μm filter) and incubating at 4°C overnight. Pellets were then collected after centrifugation and resuspended using PBS. Virus suspension was either used directly or further stored at −80°C.

For viral transduction, viruses were mixed with polybrene reagents and added to the cells. 48 h after adding the viruses, cells were incubated in the presence of 2 μg/ml puromycin for 5 days for selection. Single clones were isolated by serial dilution and cultured in 96-well plates. Positive clones were verified by fluorescence and expanded.

### CRISPR-Cas9–based generation of BLTP2 knockout and knock-in cell lines

For the BLTP2-KO cell line, the PX459 vector containing a guide RNA sequence was transfected into target cells (HeLaM). 24 h after transfection, cells were treated with 1 μg/ml puromycin for 5 days. Single cell was isolated using serial dilution and cultured in 96-well plates, then expanded in 24-well plates. Genomic DNA was extracted, and successful editing was first verified using PCR and sequencing, then further verified using western blot. The BLTP2^2xV5-KI cell line was generated by Synthego. In brief, a ribonucleoprotein with guide RNA and spCas9 was delivered with a donor sequence to the cells (HeLaM). Single clones were isolated and validated using PCR followed by sequencing. Final clones used in this study were further validated using western blot. Western blots acquisition were performed using ChemiDoc Imaging system (Bio-Rad).

### Immunoprecipitation

Immunoprecipitation of BLTP2 was performed using the Pierce IP/Co-IP kit following the procedures indicated by the manufacturer. Briefly, cells were first washed with PBS and then directly lysed by adding ice-cold IP lysis buffer to the culture dish (500 μl buffer per one 10-cm dish). Lysed cells were scraped and transferred into a 1.7-ml tube. Cells were further lysed under rotation at 4°C for 30 min. Cell lysate was clarified using a tabletop centrifuge at 17,000 *g* for 30 min 8 μg of BLTP2 antibody was added to the supernatant and incubated overnight while rotating at 4°C. The immuno-complex was then enriched using protein A-G–coated magnetic beads by incubation at room temperature for 2 h. The beads were washed two times with IP lysis buffer followed by one wash in H_2_O. Bound proteins were then eluted with “elution buffer” (pH 2.0) and neutralized with “neutralization buffer” (pH 8.5). After mixing with SDS loading buffer, samples were directly processed for SDS-PAGE without heating.

### Microscopy

All imaging experiments were performed using either a CSU-W1 microscope with Sora camera (Nikon) or a Dragonfly spinning-disk confocal imaging system with Zyla CMOS camera (Andor) with 60×/1.4 oil objective lenses. For live imaging, cells were maintained in DMEM at 37°C and 5% CO_2_ during imaging. In cells involving Halo constructs, Janelia Fluor HaloTag ligands were added to the culture medium at 1:4,000 dilution for 10 min. This medium was then replaced by fresh medium not containing Halo ligand before imaging. For immunofluorescence microscopy, cells were fixed using 4% PFA for 10 min at room temperature. Fixed cells were then permeabilized and blocked using 0.05% saponin in 10% FBS for 1 h and incubated with primary antibody at 4°C overnight, followed by secondary antibody at room temperature for 1 h. Finally, cells were mounted using ProLong Gold Antifade Mountant and kept at 4°C before imaging.

To stimulate macropinocytosis, cells were transfected with HRas G12V and imaged 48 h after transfection. In some experiments, the medium was replaced with fresh medium containing PM dye (CellBrite) (1:4,000) during imaging. Fluorescently labeled MHC-I antibodies were diluted in cell culture medium (final concentration 20 μg/ml), which was then added to the cells. Cells were imaged live by confocal microscopy within 30 min.

### Manipulations to deplete PI4P or PI(4,5)P_2_ at the PM

To reduce PI4P in the PM, the PI4KIIIα inhibitor A1 was diluted into pre-warmed culture medium to a concentration of 200 nM, then added to the cells at a 1:1 ratio at the start of the imaging session (final concentration 100 nM). For the recovery, 1 h after A1 treatment, the culture medium was removed and cells were washed twice in PBS, then changed into fresh medium for further imaging.

To acutely deplete PI(4,5)P_2_, cells were transfected with a plasmid encoding M1R with no fluorescent tag (M1R-blank). The M1R ligand Oxo-M diluted in culture medium to a concentration of 20 μM was added to cells after the start of live imaging at a 1:1 ratio (final concentration at 10 μM). Eight mins after the addition of Oxo-M, the M1R antagonist atropine (final concentration at 50 μM) was added to reverse the stimulation.

### CLEM

For CLEM, HeLaM cells were plated on a 35-mm MatTek dish (P35G-1.5-14-CGRD) and transfected as described above with plasmids encoding BLTP2^Halo and Mito-BFP. Cells were fixed with 4% PFA in PBS, then washed three times with PBS before being analyzed by fluorescence light microscopy imaging. Regions of interest were selected, and their coordinates on the dish were identified using phase contrast. Cells were further fixed with 2.5% glutaraldehyde in 0.1 M sodium cacodylate buffer, postfixed in 2% OsO_4_ and 1.5% K_4_Fe(CN)_6_ in 0.1 M sodium cacodylate buffer, *en bloc* stained with 2% aqueous uranyl acetate, dehydrated, and embedded in Embed 812. Cells of interest were relocated based on the pre-recorded coordinates. Ultrathin sections (50–60 nm) were observed in a Talos L 120C TEM microscope at 80 kV; images were taken with Velox software and a 4k × 4k Ceta CMOS Camera (Thermo Fisher Scientific).

### Image processing and statistical analysis

Image processing (including deconvolution, image smoothing, ROIs creation, and fluorescence intensity quantification) was performed using either ImageJ (Fiji) or the Nikon Elements software package with built-in plugins. Statistical analysis was performed using Prism 10 (GraphPad).

#### Quantification of the BLTP2 fluorescence in phosphatase recruitment assays

Related to Fig. 3, C–E: for each condition, cells were recorded live for 20–25 min after the addition of rapamycin at time zero. ROIs corresponding to BLTP2-positive tubular structures were selected manually in the first and last frame of each movie using the free-hand drawing tool in ImageJ. The total BLTP2 fluorescence intensity of these structures from each cell was then measured in ImageJ, and the change in fluorescence was quantified by dividing the fluorescence intensity in the last frame by the fluorescence intensity in the first frame.

Related to Fig. 5, A–D: for each condition, cells were recorded live for 20–25 min after the addition of rapamycin at time zero. Using the free-hand drawing tool in ImageJ, a straight line was drawn perpendicular to a same spot of the PM in first and the last frame of each movie. The peak of the C2_FAM102A_-EGFP or C2_FAM102B_-EGFP florescence intensity of these lines (corresponding to the PM) was measured, and the change in fluorescence was quantified by dividing the peak intensity in the last frame by the peak intensity in the first frame.

#### Quantification of BLTP2 mutants’ enrichment at ER-PM contacts relative to the global ER

Related to Fig. 4 L: The enrichment of FL BLTP2 (BLTP2^EGFP) and of BLTP2^Halo-△FBM in the cortical ER relative to the bulk of the ER was calculated by dividing their fluorescence in an arbitrary ROI of the basal surface of the cell by their fluorescence in an equivalent ROI in a roughly equatorial regions of the same cell. Then, the enrichment ratio was quantified by dividing the enrichment of BLTP2^EGFP by the enrichment of BLTP2^Halo-△FBM.

#### Quantification of BLTP2 mutants’ enrichment on tubular structures

Fig. 6, D–F, K, and L: cells co-expressing FL BLTP2 (BLTP2^EGFP) and BLTP2 mutants (BLTP2^Halo-△FBM, BLTP2^Halo^RP→AA^ and BLTP2^Halo^RP→AA^-△FBM) were used for this analysis. Using the free-hand drawing tool of image J, ROIs corresponding to the entire area of each cells (ROI-1) or to all the tubular structures positive for FL BLTP2^EGFP in the same cells (ROI-2) were selected. Next, the average EGFP and Halo fluorescence on both ROIs was measured, and the ratio between the average fluorescence intensity of ROI-2 and ROI-1 was calculated and defined as the “Halo/EGFP” enrichment ratio shown in Fig. 6, F and L.

### Online supplemental material

Fig. S1 shows the BLTP2 localization in different cell types. Fig. S2 shows the additional data of BLTP2’s targeting to the PM and PM-connected tubules. Fig. S3 shows the additional data of the BLTP2 interaction with FAM102A and FAM102B. Fig. S4 shows the BLTP2 being recruited to mAmph2-positive tubular structures in COS-7 cells. Fig. S5 shows the additional data on BLTP2’s recruitment to macropinosomes undergoing fusion with the PM. Video 1 shows that the BLTP2 is recruited to Rab10-positive tubular endosomes when they fuse with the PM. Video 2 shows that the BTLP2 re-establishes contacts with PM-connected tubular structures after A1 washout. Video 3 shows that the BLTP2 disassociates from FAM102A-mediated ER-PM contact sites upon Oxo-M treatment. Video 4 shows that the BLTP2 disassociates from FAM102B-mediated ER-PM contact sites upon Oxo-M treatment. Video 5 shows that the BLTP2 is recruited to an MHC-I–positive macropinosome undergoing fusion with the PM. Video 6 shows the recruitment of BLTP2 to multiple MHC-I–positive macropinosomes upon their fusion with the PM. Video 7 shows that the recruitment of BLTP2 to an MHC-I–positive macropinosome follows a resurgence of the PI(4,5)P_2_ signal on the macropinosome. Video 8 shows the acquisition of a PM-associated dye signals fusion of a macropinosome with the PM. Video 9 shows that a subset of PI(4,5)P_2_-positive vacuoles accumulated in a BLTP2-KO cell is continuous with the PM. Video 10 shows that a subset of PI(4,5)P_2_-positive vacuoles accumulated in a BLTP2-KO cell is accessible to extracellular dextran, a fluid phase marker. Table S1 lists the information on key resources used in this study.

### Resource availability

#### Lead contact

Further information and requests for resources and reagents should be directed to the lead contact, Pietro De Camilli (pietro.decamilli@yale.edu).

#### Materials availability

Plasmids and all other reagents generated in this study are available upon request from the lead contact.

## Supplementary Material

Table S1 lists the information on key resources used in this study.

SourceData F1 is the source file for Fig. 1.

SourceData F8 is the source file for Fig. 8.

## Data Availability

This paper does not report original code. Any additional information is available from the lead contact upon request.
