# Supplementary figures and images for: Multiple interactions recruit BLTP2 to ER-PM contacts to control plasma membrane dynamics
Source: J Cell Biol. 2025 Sep 3;224(11):e202504027. doi: 10.1083/jcb.202504027 (PMC12406788; doi:10.1083/jcb.202504027)

Figure 1G

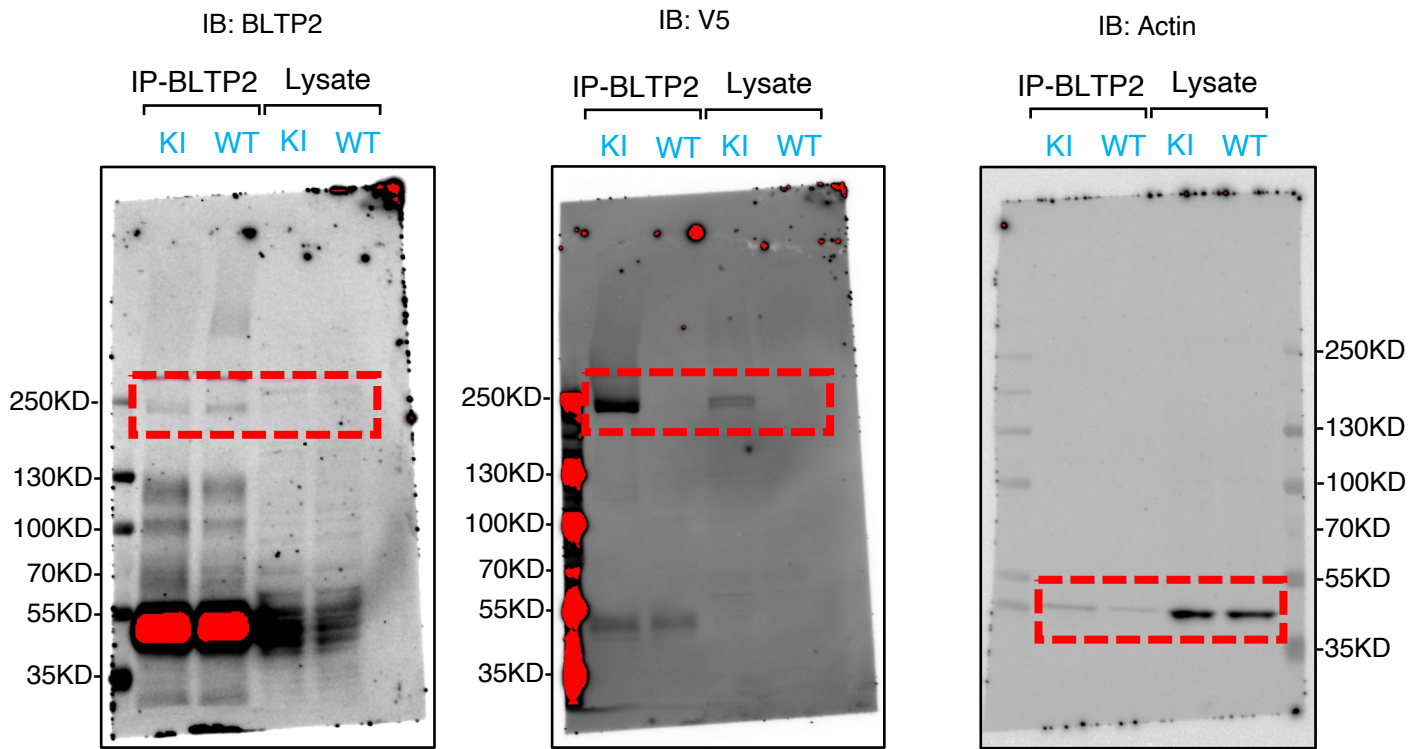

Supplement: SourceData F1 — is the source file for Fig. 1. [file jcb_202504027_sourcedataf1.pdf]

Figure 8A

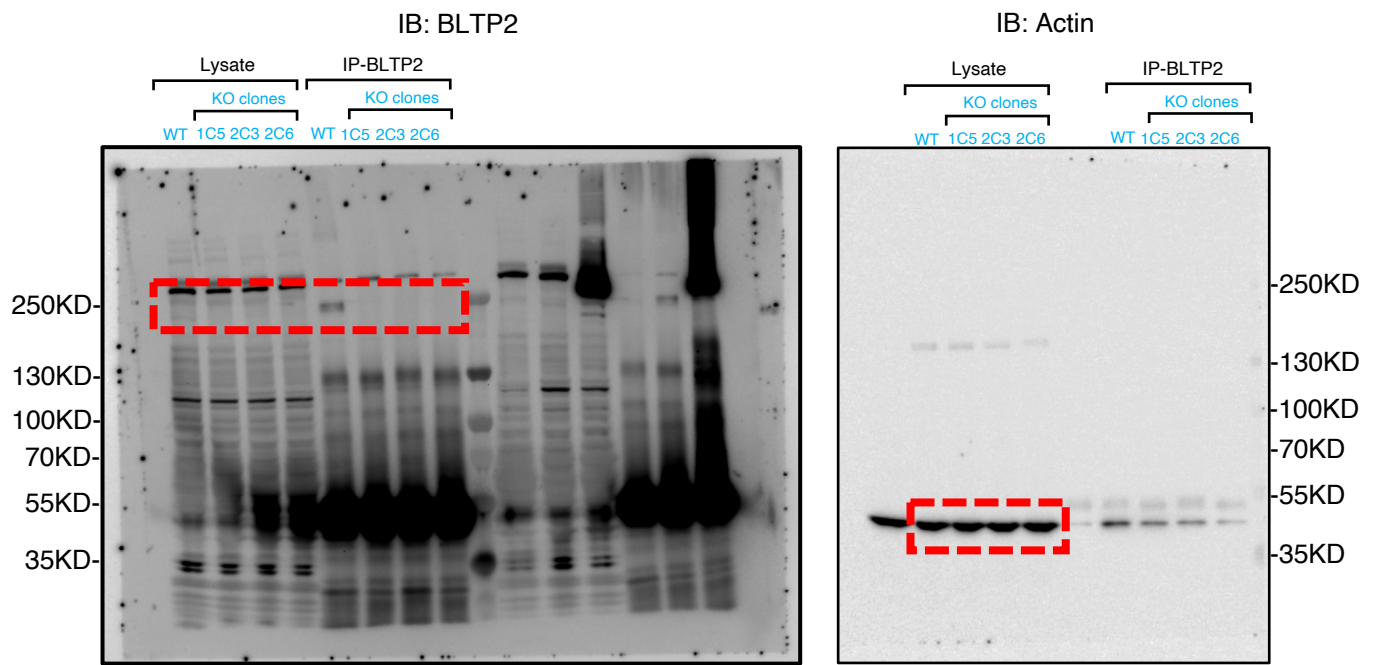

Supplement: SourceData F8 — is the source file for Fig. 8. [file jcb_202504027_sourcedataf8.pdf]
